# Advances in the construction of diverse SuFEx linkers

**DOI:** 10.1093/nsr/nwad123

**Published:** 2023-04-29

**Authors:** Daming Zeng, Wei-Ping Deng, Xuefeng Jiang

**Affiliations:** Shanghai Frontiers Science Center of Optogenetic Techniques for Cell Metabolism, School of Pharmacy, East China University of Science and Technology, Shanghai 200237, China; Shanghai Key Laboratory of Green Chemistry and Chemical Process, School of Chemistry and Molecular Engineering, East China Normal University, Shanghai 200062, China; Shanghai Frontiers Science Center of Optogenetic Techniques for Cell Metabolism, School of Pharmacy, East China University of Science and Technology, Shanghai 200237, China; Shanghai Key Laboratory of Green Chemistry and Chemical Process, School of Chemistry and Molecular Engineering, East China Normal University, Shanghai 200062, China

**Keywords:** SuFEx, SuFEx linker, S^VI^–F bond, synthetic strategy

## Abstract

Sulfur fluoride exchange (SuFEx), a new generation of click chemistry, was first presented by Sharpless, Dong and co-workers in 2014. Owing to the high stability and yet efficient reactivity of the S^VI^–F bond, SuFEx has found widespread applications in organic synthesis, materials science, chemical biology and drug discovery. A diverse collection of SuFEx linkers has emerged, involving gaseous SO_2_F_2_ and SOF_4_ hubs; SOF_4_-derived iminosulfur oxydifluorides; *O*-, *N*- and *C*-attached sulfonyl fluorides and sulfonimidoyl fluorides; and novel sulfondiimidoyl fluorides. This review summarizes the progress of these SuFEx connectors, with an emphasis on analysing the advantages and disadvantages of synthetic strategies of these connectors based on the SuFEx concept, and it is expected to be beneficial to researchers to rapidly and correctly understand this field, thus inspiring further development in SuFEx chemistry.

## Teaser

SuFEx linkers are of great importance in the sulfur-fluoride cleavage and linkage chemistry, and their development and synthesis have been discussed in this review.

## INTRODUCTION

**Figure 1.**
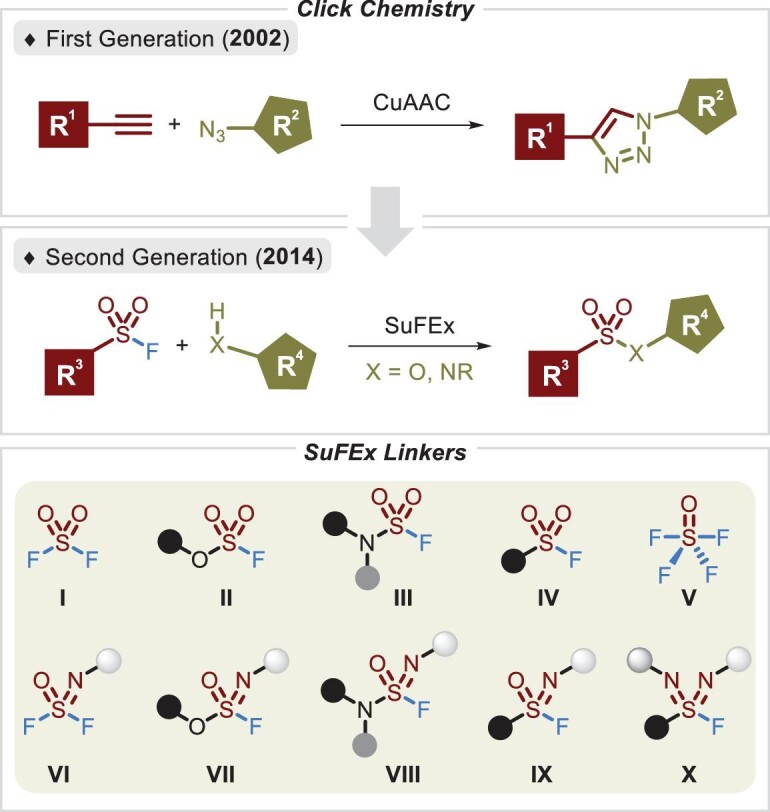
Two generations of ideal click chemistry reactions and an available series of SuFEx linkers.

Click chemistry [1], also known as linking chemistry, provides simple and efficient access to a diversity of molecules via the assembly of small units, with a predominance of carbon–heteroatom linkages. Notably, Cu-catalysed alkyne–azide cycloaddition (CuAAC), as the first generation of such reactions [2], has been widely applied in various fields and has recently become one of the most useful synthetic strategies. Based on the balance between the distinctive reactivity and favorable stability of the S^VI^–F bond, a new generation of click chemistry, sulfur(VI) fluoride exchange (SuFEx), was first established by Sharpless *et al*. in 2014 [3], implementing the preparation of hypervalent sulfur compound libraries under metal-free conditions. The S^VI^–F motif-containing compounds impart desired properties such as resistance to hydrolysis and thermolysis; stability toward acids, bases, redox conditions, light and other routine reaction conditions; and controllability and specificity of the S–F bond cleavage [4,5]. In this vein, the installation and modification of sulfur(VI) fluorides are greatly flexible. However, the S^VI^–Cl bond-containing molecules are unstable in moist or hot atmospheres, and because of the lower electronegativity and higher polarizability of the chloride atom relative to the fluoride atom, their electrophilic sites tend to be the chloride atoms, especially with carbon nucleophiles [3]. The importance of the S^VI^–F bond has been gradually elucidated and a series of SuFEx linkers have been developed. As shown in Figure 1, SO_2_F-containing compounds (**I**–**IV**) are frequently utilized for the construction of multifunctional substrates via further derivatization [5,6]. The mono-aza analogs of sulfonyl fluorides (**VI**–**IX**), by contrast, have only recently attracted considerable attention. Unlike common sulfur(VI) fluoride compounds, the mono-aza analogs of sulfonyl fluorides have an additional nitrogen atom, which serves as an additional site for modification, thereby tuning their chemical stability and reactivity [3,4]. Of great interest is the construction of sulfondiimidoyl fluorides (**X**), which themselves feature two S=N bonds and present tremendous potential [7].

**Figure 2.**
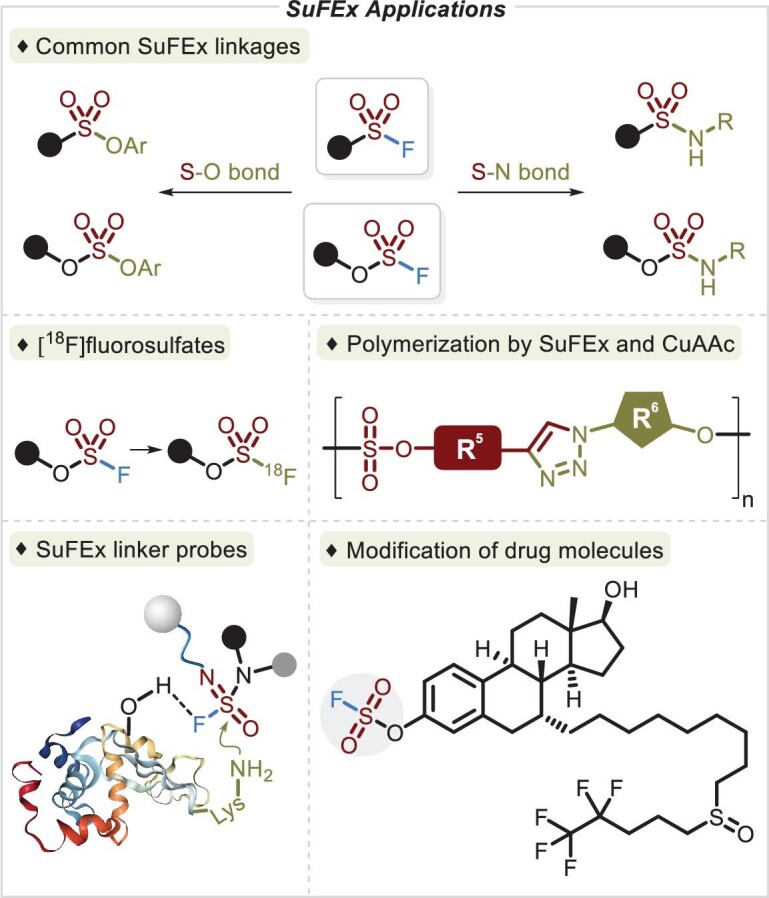
SuFEx applications involving organic synthesis, polymer chemistry, chemical biology and drug discovery via a library of SuFEx linkers.

The unique and favorable properties and available synthesis methods of S(VI) fluoride compounds substantially facilitate the application of these linkers in organic synthesis [8–13], polymer chemistry [14–17], chemical biology [18–22] and drug discovery [23,24] (Figure 2). Generally, SuFEx connectors, as appealing electrophiles, are deployed in the construction of organosulfur(VI) derivatives, primarily for the linkage of S–O and S–N bonds [3,8–10], and unprecedented [^18^F]-labeled fluorosulfates within 1 minute [13]. The SuFEx reaction, characterized by rapid reactivity, a high yield and expedient purification, is potentially suitable for polymer assembly. An abundance of polysulfates, polysulfonates and their aza variants have been successfully generated via the SuFEx strategy [14,16] and they exhibit greater chemical durability and more favorable mechanical characteristics than well-known polycarbonates. Additionally, orthogonal SuFEx and CuAAC reactions were used to assemble sequence-regulated synthetic polymers by one-pot polymerization of SuFExable monomers [15] and sequential click reactions afforded branched functional polymers via post-modification of SOF_4_-derived copolymers [16]. The hydrolytic stability of the S^VI^–F bond plays a crucial role in chemical biology, ensuring that the hexavalent sulfur fluoride precursor is unreactive until the S^VI^–F bond is activated by a protein via hydrogen bonding [4]. The sulfur(VI) fluoride compound is installed so that it will be close to nucleophilic residues, such as lysine, histidine and tyrosine, thus enabling the SuFEx process [18]. By positioning the S^VI^–F fragment on drug molecules, the resulting compounds often show improved bioactivity along with better physicochemical and pharmacokinetic profiles [23].

In this review, we discuss SuFEx linkers (Figure 1, **II**–**IV** and **VI**–**X**) in terms of their properties, reaction routes, substrate scopes and mechanisms, as well as the two gases, sulfuryl fluoride (SO_2_F_2_, **I**) [25] and thionyl tetrafluoride (SOF_4_, **V**) [26]. The synthetic protocols reported after 2014 are elaborated on, highlighting their advantages and disadvantages. Although several reports involving the SuFEx chemistry of sulfonyl fluorides (**IV**) [5,27] and the growing application of modular SuFEx hubs **(I, IV** and **V**) have been reviewed [6], we focus on the assembly of these SuFEx connectors (**II–IV** and **VI–X**) and hope to deliver an up-to-date review for ease of rapid acquisition and extensive application.

## FLUOROSULFATES (ROSO_2_F, II)

Fluorosulfate acts as not only a SuFExable substrate but also a leaving group analogous to organic halides and triflates. When alcohols fuse with the -SO_2_F fragment, the *ipso*-carbon of the resulting alkyl fluorosulfates is typically liable to be attacked by nucleophiles [28]. However, aromatic fluorosulfates derived from phenols are more stable than alkyl-SO_2_F groups. They can remain stable for several months in a neutral solution and do not react with monomeric natural amino acids on denatured proteins [3], despite their excellent triflate-like activity in metal couplings [29]. Therefore, strategies to generate aromatic fluorosulfates are of considerable interest.

**Figure 3.**
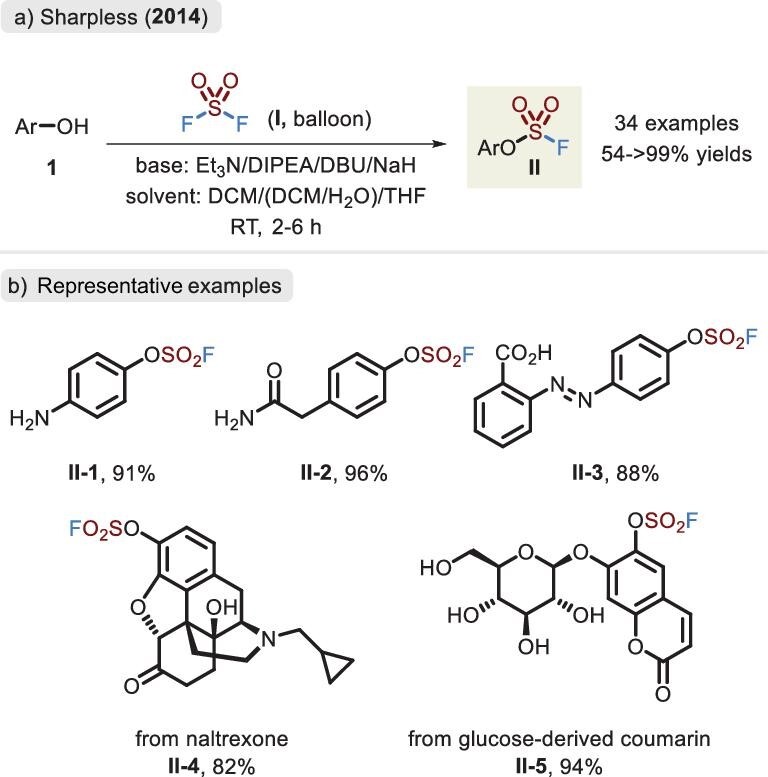
(a) Synthesis of aromatic fluorosulfates by phenols and SO_2_F_2_ via the optimized conditions by Sharpless and co-workers. (b) Representative examples of fluorosulfate.

The first synthesis of aromatic fluorosulfates was documented by Lange and Müller in 1930 [30] and proceeded via the thermolysis of arenediazonium fluorosulfates and the release of a molecule of N_2_. Subsequently, some research groups developed a strategy in which commercially available phenols reacted directly with SO_2_F-containing electrophiles such as FSO_2_Cl, FSO_2_OSO_2_F, HOSO_2_F and SO_2_F_2_ [31]. While rapid and convenient, this method required harsh conditions (e.g. strong acid), demonstrating poor functional-group tolerance. With a wide range of sources and a low price, SO_2_F_2_ is an appealing SuFEx linker that has successfully been applied in the assembly of fluorosulfates with almost quantitative yields by Sharpless and co-workers (Figure 3a) [3]. This method showed favorable substrate scope and was compatible with alcohol hydroxyl, arylaminyl and carboxyl groups (Figure 3b). Interestingly, for several complicated architectures, the addition of water was beneficial to suppress the competitive reactions of other nucleophilic groups, partly due to the productive hydrogen-bond interactions. In this manner, the reaction of Fmoc-protected tyrosine with SO_2_F_2_ proceeded well in a biphasic mixture (saturated borax/DCM) [32].

**Figure 4.**
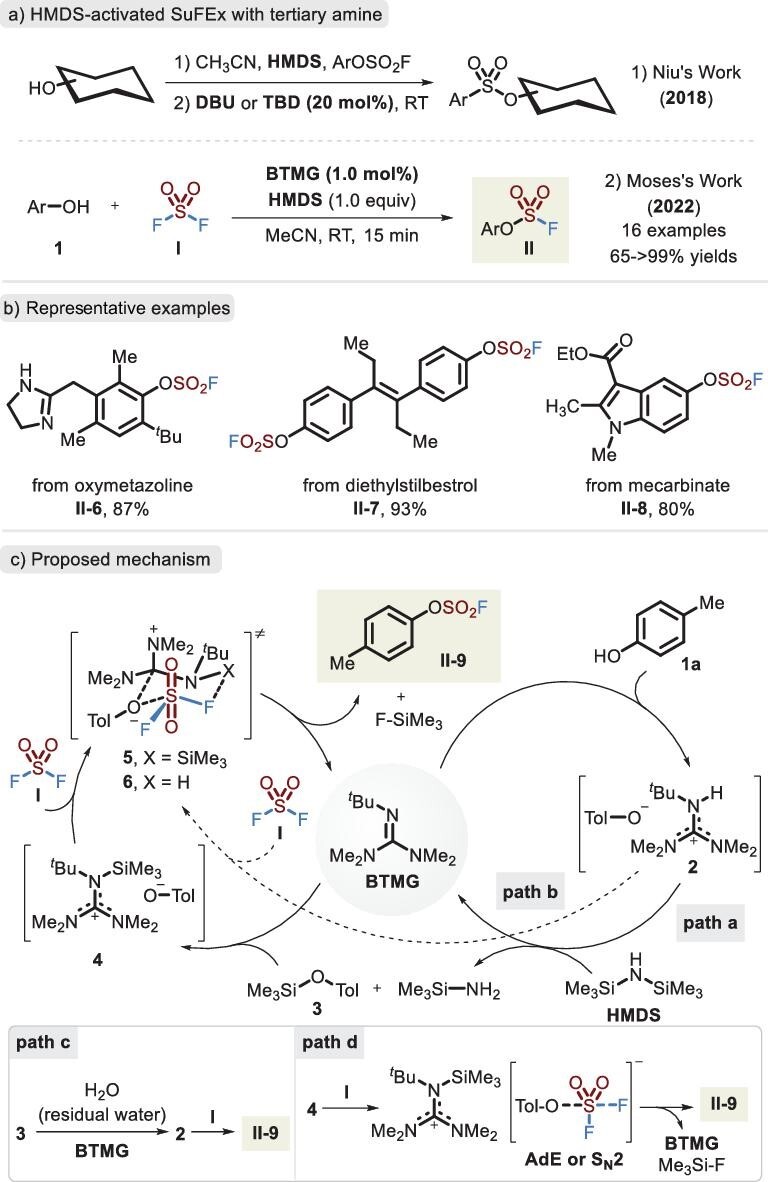
(a) Accelerated SuFEx reaction for assembling the fluorosulfates via the BTMG catalyst and HMDS additive. (b) Representative examples of fluorosulfate. (c) Proposed mechanism involving paths a and b, adapted from [9].

A remarkable advance in the use of the SuFEx strategy for the construction of S–O bonds was developed by Niu and co-workers, who employed hexamethyldisilazane (HMDS) as a hydroxyl-activated reagent and DBU or 1,5,7-triazabicyclo[4.4.0]dec-5-ene (TBD) as a catalyst to realize the *O*-sulfation of saccharides via a one-pot procedure [33] (Figure 4a, reaction 1). In 2022, Moses *et al.* described an analogous method [9] in which the addition of an extra silicon reagent (HMDS) not only activated the phenolic hydroxyl, but also scavenged the generated hydrogen fluoride (HF) without excess tertiary amines (Figure 4a, reaction 2). Only a low load of BTMG (2-*tert*-butyl-1,1,3,3-tetramethylguanidine, 1 mol%) was used as a SuFEx catalyst within a short reaction time (15 min), effectively achieving S–O bond construction. A mechanism, accordingly, was proposed, as shown in Figure 4c. Initially, the reaction is initiated via the deprotonation of **1a** by BTMG to generate complex **2**, rather than the interaction [34] between BTMG and SO_2_F_2_. Guanidinium salt **4** is liberated by HDMS, followed by a strong attraction between Si and F (BDE = 135 kcal/mol) [8], thus enhancing the electrophilicity of the sulfur center and affording fluorosulfate **II-9** via a possible six-membered transition state **5** (path a). Alternatively, the hydrogen-bond interaction of guanidinium salt **2** and SO_2_F_2_ in transition state **6** may also facilitate the generation of product **II-9** (path b). In addition to the abovementioned process, we propose that intermediate **3** can react with residual water to deliver key complex **2** in the presence of BTMG, followed by transition state **6** to construct the S–O bond (path c). Due to the steric hindrance of intermediate **4**, the chair-like transition state **6** is difficult to access; therefore another possible stepwise route is preferentially involved (path d). The reaction of phenol anion with SO_2_F_2_ is occurred via an AdE (addition–elimination) or S_N_2 process, and subsequently the releasing fluoride anion induces the removal of TMS.

**Figure 5.**
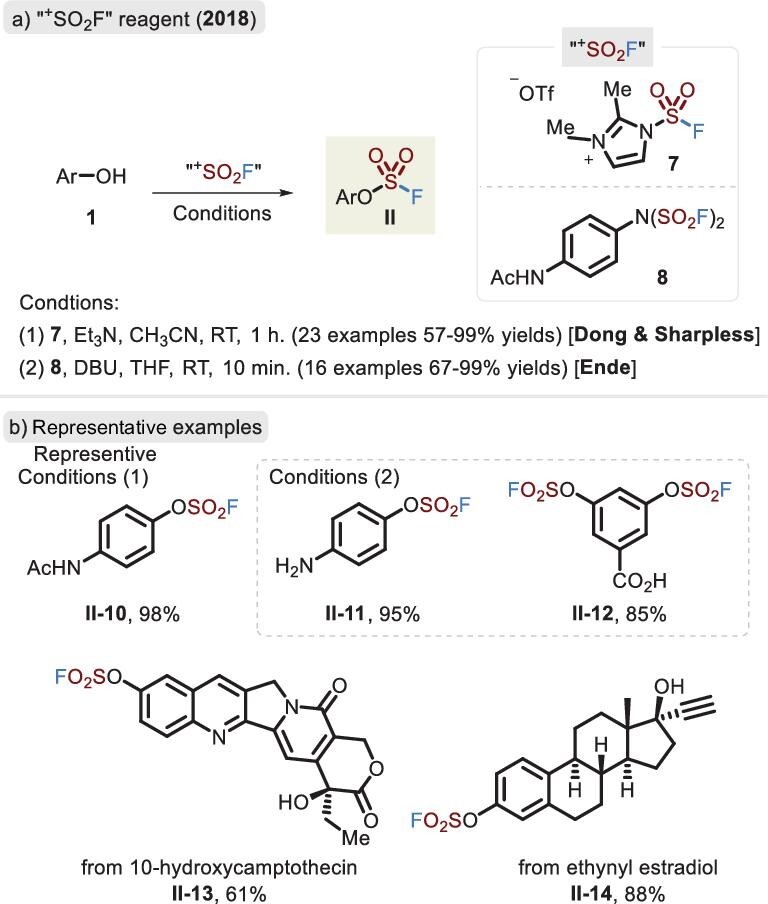
(a) Modular synthesis of fluorosulfates via the solid SO_2_F_2_ surrogates, FSITs and AISF. (b) Representative examples realized via different reaction conditions.

Although gaseous SO_2_F_2_ is commercially available and ubiquitously applied as a fumigant, it is toxic and corrosive [25]. Thus, the inability of apparatuses to handle harmful gas and the limination of regulations to obtain SO_2_F_2_ are obstacles to the application of this approach in some research groups. Accordingly, it is essential that a stable and easy-to-handle SO_2_F_2_ surrogate be developed to access SuFExable compounds. In 2017, Borggraeve and co-workers utilized 1,1’-sulfonyldiimidazole (SDI) as a precursor in combination with potassium fluoride (KF) and trifluoroacetic acid (TFA) to release SO_2_F_2_ gas within a two-chamber reactor [35]. The substitution of SO_2_F_2_ with a broad range of phenols was implemented with satisfactory yields by Sharpless's method (Figure 3a). However, the specialized reactor was inconvenient for use by researchers. To address this challenge, solid fluorosulfuryl imidazolium triflate salt **7** (FSITs) was synthesized to afford the fluorosulfonyl moiety by the research group of Dong and Sharpless in 2018, and this compound demonstrated better reactivity than SO_2_F_2_ and favorable chemoselectivity (Figure 5a, condition 1) [36]. The reaction showed a pronounced functional-group tolerance with the aliphatic hydroxyl group retained and was successfully applied in the last-stage modification of pharmaceuticals and natural products. In the same year, the Ende group also reported a novel SO_2_F_2_ surrogate, [4-(acetylamino)phenyl] imidodisulfuryl difluoride **8** (AISF), which rapidly provided a wealth of fluorosulfates with the promotion of superstoichiometric DBU (Figure 4a, condition 2) [37]. AISF, in contrast to FSITs, is shelf-stable, even dissolving in solvents with no obvious decomposition for more than a month. In this regard, unprotected amino and carboxyl groups may selectively lead to the corresponding fluorosulfates in good yields (Figure 5b, **II-11** and **II-12**).

## SULFAMOYL FLUORIDES (R^1^R^2^NSO_2_F, III)

Sulfamoyl fluorides exhibit distinct properties, which are entirely attributed to their nitrogen fragments. Primary amines couple with SO_2_F_2_ and the resulting compounds contain an N–H moiety, which themselves are unstable in the presence of bases and easily eliminate HF to release more electrophilic azasulfenes [38]. Methods for the construction of monosubstituted sulfamoyl fluorides were therefore developed under acidic and neutral conditions [39] but suffer from narrow substrate scopes. In contrast, the secondary-amine-derived sulfamoyl fluorides manifest notable stability and remain stable even upon exposure to harsh conditions (such as strong bases and reductants), indicating that the further SuFEx reaction of *N*-disubstituted sulfamoyl fluorides, to a certain extent, is greatly impeded. Fortunately, this challenging process can proceed smoothly via the activation of Lewis acids [40].

**Figure 6.**
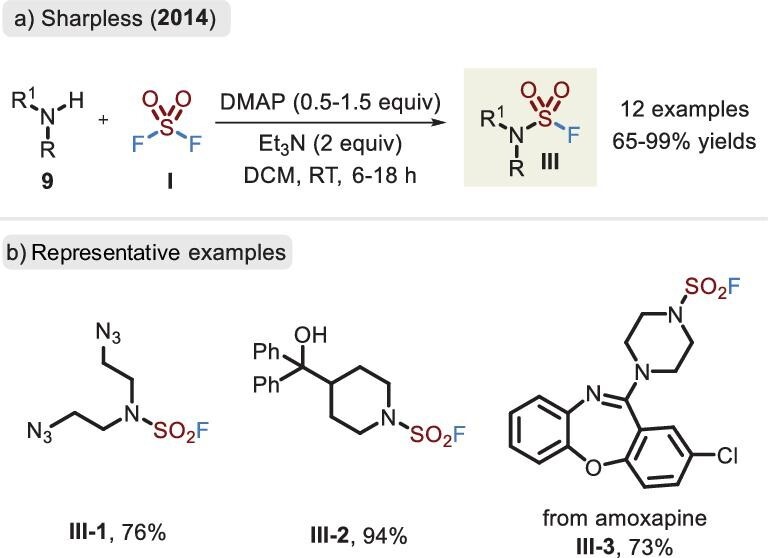
(a) Synthesis of disubstituted sulfamoyl fluorides via SO_2_F_2_ and amines with the aid of DMAP. (b) Representative examples of sulfamoyl fluoride.

Similar to the assembly of flurosulfates, the reaction of SO_2_F_2_ with disubstituted amines was effectively performed by Sharpless *et al*. in 2014 (Figure 6a) [3]. An activating reagent, 4-Dimethylaminopyridine (DMAP), was necessary in the reaction system. Sensitive functional groups, including aliphatic hydroxyl, alkenyl, alkynyl, azide, etc., were found to be compatible. Unfortunately, secondary anilines with poor nucleophilicity could not afford the corresponding sulfamoyl fluorides.

**Figure 7.**
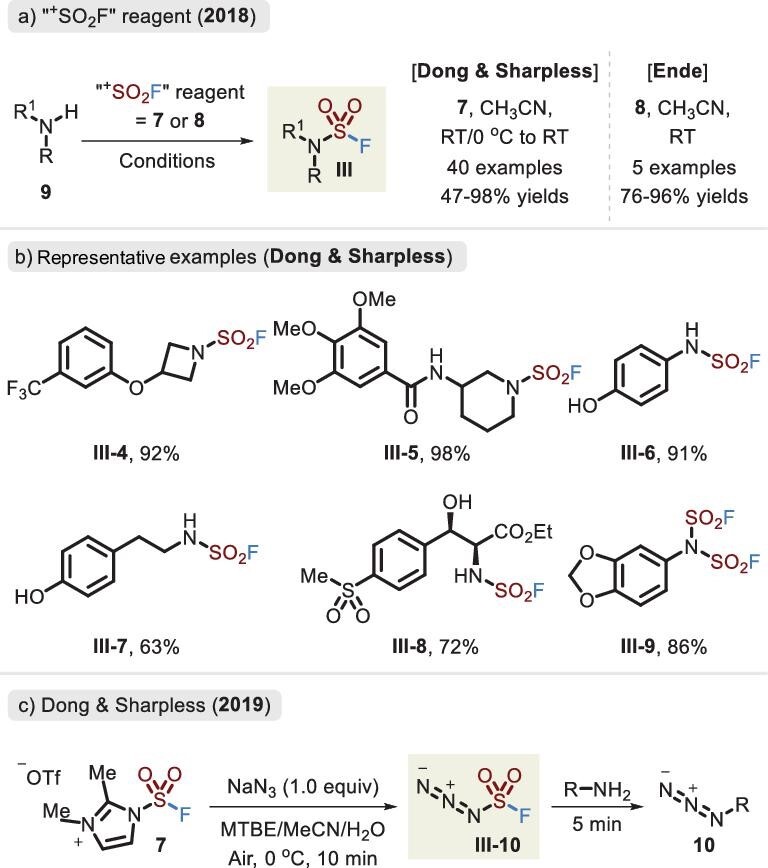
(a) Synthesis of the sulfamoyl fluorides via the two solid surrogates. (b) Representative examples of Dong's method. (c) Synthesis and application of fluorosulfuryl azide.

In 2018, the groups of Dong and Sharpless [36] and Ende [37] independently developed a solid ‘^+^SO_2_F’ precursor, which successfully realized not only the synthesis of flurosulfates via phenols (Figure 5a) but also the linkage of S–N bonds to provide sulfamoyl fluorides (Figure 7a). Notably, FSITs (**7**) led to higher reactivity than SO_2_F_2_ and AISF (**8**) and resulted in a good substrate scope, including the previously restricted anilines and primary amines. As demonstrated in Figure 3b, the basic environment caused preferential reaction of the phenol rather than aniline. However, when FSITs was attacked by bifunctional nucleophiles (comprising amino and hydroxyl groups), the reaction afforded sulfamoyl fluorides in the absence of a base, retaining the hydroxyl fragments (Figure 7b, **III-6** to **III-8**). In addition, bis(fluorosulfuryl)imides were accessible in favorable yields.

During the search for a new SuFExable linker, the safe and effective synthesis of fluorosulfuryl azide **III-10** was unexpectedly discovered by the Dong and Sharpless group in 2019 (Figure 7c) [41]. Interestingly, the reagent did not undergo the expected SuFEx reaction; rather, it revealed unusual diazotransfer reactivity toward primary amines. A considerably wide range of substrates (>1000 amines) were accommodated in this reaction, which offered a safe, practical and modular route to access azide compounds.

**Figure 8.**
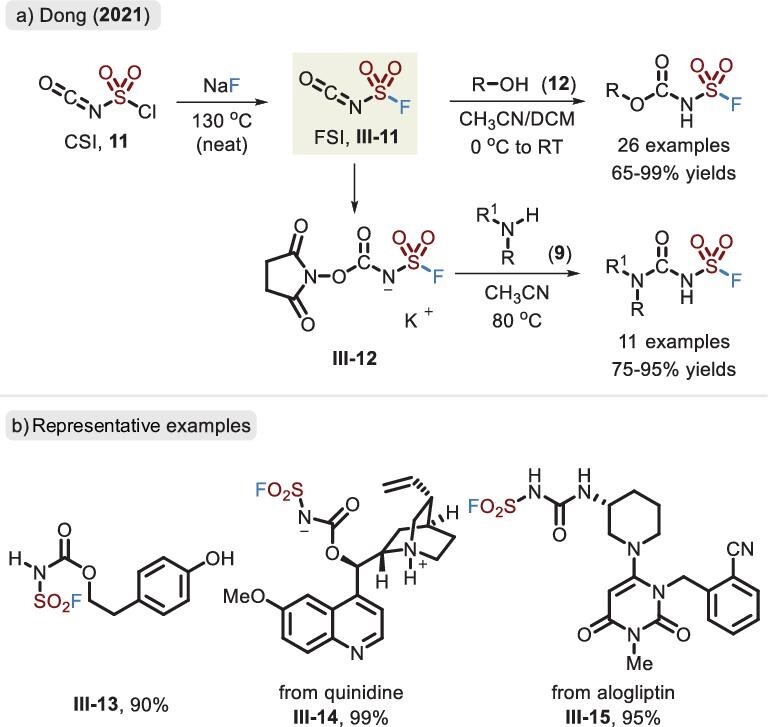
(a) Synthesis of FSI via CSI and derivatization based upon the alcohols and amines. (b) Representative examples of fluorosulfuryl carbamate and urea.

In 2021, Dong *et al.* extended the accessible chemical space for the identification of *N*-amide-derived sulfamoyl fluorides, where fluorosufuryl isocyanate (FSI, **III-11**) played a vital role (Figure 8a) [42]. It originated from the halogen exchange of chlorosulfuryl isocyanate (CSI, **11**), as a reliable linker, allowing the installation of alcohols and amines. Unlike the SuFEx process, the N=C=O fragment of FSI was preferentially attacked by aliphatic alcohols over phenols. Noticeably, the ester-based sulfamoyl fluoride, relative to its chloride, remained stable in the form of a salt (**III-12**), without obvious elimination, and served as a novel precursor of *N*-fluorinated imide for forging the C–N bond [43].

## SULFONYL FLUORIDES (RSO_2_F, IV)

**Figure 9.**
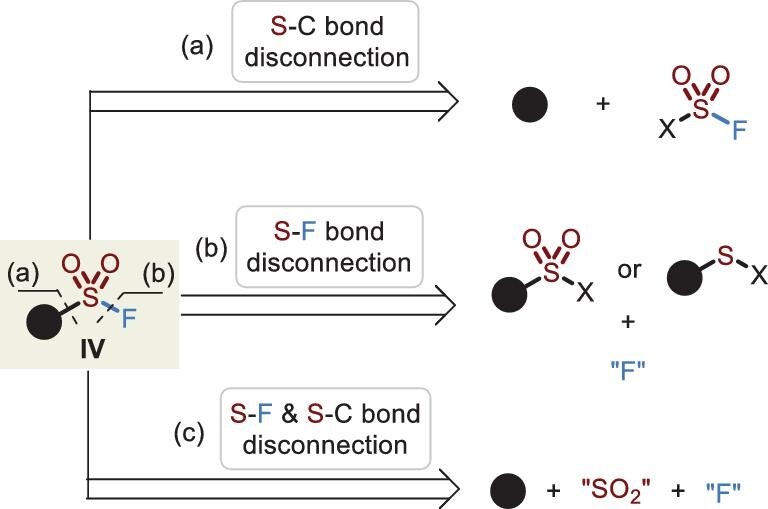
Strategies for the synthesis of sulfonyl fluorides presented by three bond-disconnection methods: (a) S–C bond disconnection; (b) S–F bond disconnection; (c) S–F and S–C bonds disconnection.

Unlike the formation of fluorosulfates (or sulfamoyl fluorides) by rigidly fusing SO_2_F_2_ with phenols (or amines), strategies for the synthesis of sulfonyl fluorides are relatively flexible and abundant [6,27]. In light of different bond-disconnection forms, the strategies are classified into three categories as demonstrated in Figure 9, among which cleavage of the S–F bond (path b) is the most common and practical protocol for researchers to access these compounds. A three-component-coupling strategy (path c) via the insertion of sulfur dioxide for the construction of sulfonyl fluorides has been quite attractive, due to the untrammeled varying of carbon-linked moieties, avoiding the use of unpleasant thiols (path b). Notably, the development of fluorosulfonyl-containing solid reagents effectively realizes the assembly of S–C bonds (path a). Moreover, photocatalytic and electrochemical conditions also allow the effective installation of the -SO_2_F fragment. A summary of this new sulfonyl fluoride assembly will be presented, highlighting a selection of new methods [27].

### Disconnection of S–C bond

#### ‘^+^SO_2_F’ synthon

The attachment of the fluorosulfonyl motif to arenes employing the ‘^+^SO_2_F’ precursor was preformed, analogous to the synthesis of aromatic sulfonyl chlorides via the Friedel–Crafts reaction in industry. Originally, Steinkopf first disclosed the assembly of sulfonyl fluorides using fluorosulfonyl acid (FSO_2_OH), while the regioselectivity issue was unavoidable [6]. Prefunctionalization of the carbon sites allowed access to these molecules, as independently developed by the groups of Sammis [44] and Kim [45] in 2019. Although direct and concise, this strategy encountered several limitations, such as harsh reaction conditions, restricted substrate scopes and excessive non-target products.

#### ‘^•^SO_2_F’ synthon

The FSO_2_-bearing reagent, geared toward the installation of a fluorosulfonyl core, is highly attractive for the assembly of fluorosulfates and sulfamoyl fluorides via FSITs (**7**) or AISF (**8**). These two compounds served as ‘^+^SO_2_F’ linkers to engage in this process, but unfortunately they could not implement the S–C bond connection. In 2021, the first example of radical fluorosulfonylation was disclosed by Liao and co-workers, employing sulfuryl chlorofluoride (FSO_2_Cl) as the fluorosulfonyl radical precursor with olefins to forge alkenyl sulfonyl fluorides under photoredox conditions [46]. The elusive FSO_2_^•^ species was studied through density functional theory (DFT) calculations, in comparison with the trifluoromethylsulfonyl radical (CF_3_SO_2_^•^). It was found that FSO_2_^•^ relative to CF_3_SO_2_^•^ revealed a more planar configuration and strong electrophilicity of the sulfur center, favoring the attack of alkenes.

**Figure 10.**
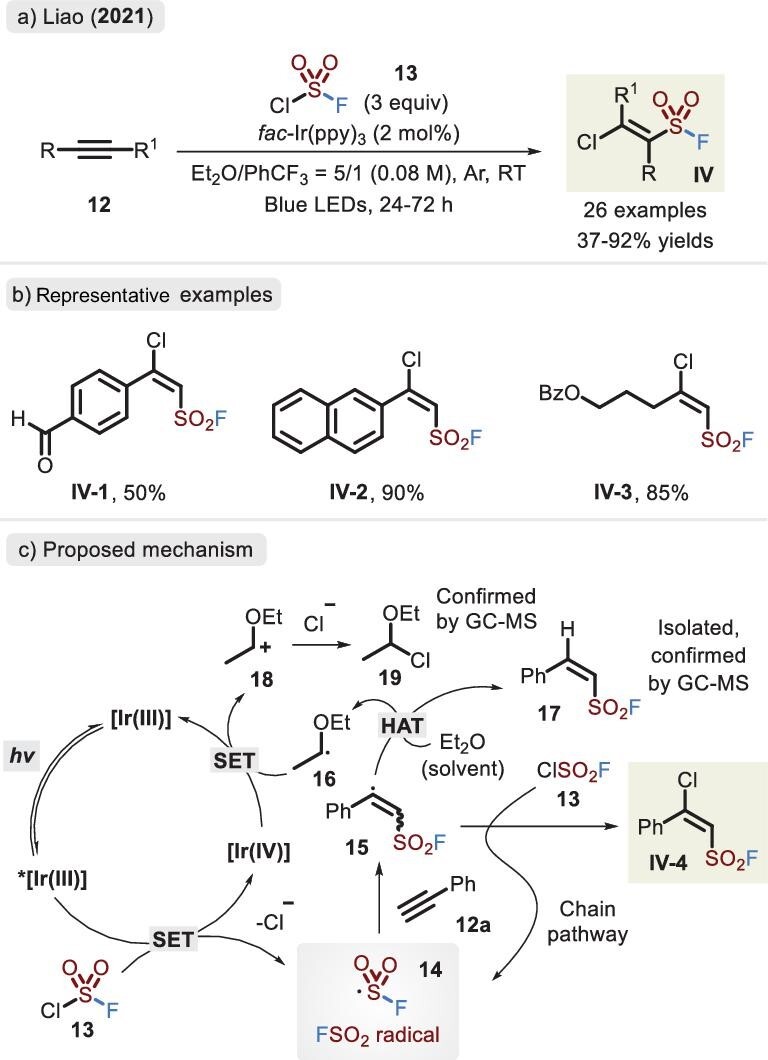
(a) Synthesis of *β-*chloro alkenylsulfonyl fluorides via the fluorosulfonyl radical under photoredox conditions. (b) Representative examples. (c) Proposed mechanism of *β-*chloro alkenylsulfonyl fluoride, adapted from [47].

Likewise, *π* electrons of alkynes efficaciously overlapped the *p*-orbital of liberated fluorosulfonyl radical, which was developed by the same group (Figure 10a) [47]. An abundance of *β*-chloro-derived alkenylsulfonyl fluorides (BCASFs) comprising aryl, thienyl and alkyl motifs were furnished. Furthermore, the chloride site of BCASFs was further modified via transition-metal-catalysed coupling and nucleophilic substitution with the S–F bond untouched, which has remarkably enriched the SuFEx combinatorial library. A plausible mechanism was described based on the experimental results (Figure 10c). Fluorosulfonyl radical **14** is first generated via the single-electron transfer (SET) of excited iridium catalyst *[Ir(III)], followed by the trapping of alkyne **12a** to liberate radical species **15**, which eventually reacts with sulfuryl chlorofluoride **13** to deliver the corresponding product **IV-4**. Moreover, the solvent Et_2_O plays a crucial role in the regeneration of the photocatalyst ([Ir(IV)] to [Ir(III)]), occurring in the hydrogen atom transfer (HAT) and SET processes.

**Figure 11.**
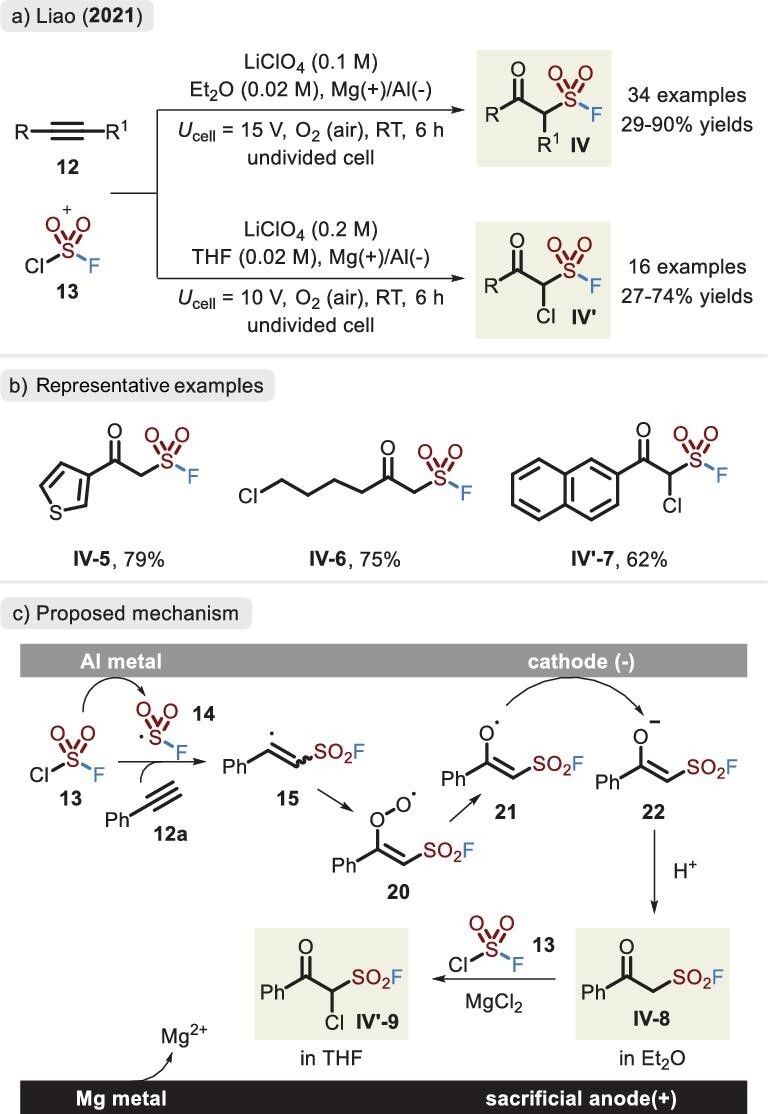
(a) Divergent *oxo*-fluorosulfonylation of alkynes via FSO_2_Cl under electrochemical conditions. (b) Representative examples. (c) Proposed mechanism for the *β*-keto sulfonyl fluorides and *α*-chloro-*β*-keto sulfonyl fluorides, adapted from [48].

The introduction of fluorosulfonyl radical **14** via electrochemical conditions is also a good choice with unexpected consequences. Liao's group first realized the electrochemical *oxo*-fluorosulfonylation of alkynes with air as the oxidant, allowing access to *β*-keto sulfonyl fluorides **VI** in moderate to good yields (Figure 11a) [48]. Sensitive motifs, such as aldehyde-, alcohol- and carboxylic acid-substituted substrates, were equally competent in this transformation. Interestingly, subtle changes involving an increase in electrolyte concentration and variation in solvent resulted in divergent products. Excessive MgCl_2_ exhibited higher solubility in tetrahydrofuran, which itself as a Lewis acid facilitated *α*-chloro ligation and thus released compound **VI’**. Mechanistic studies demonstrated that O_2_ is the only oxygen source and couples with radical **15** (Figure 11c). Alternatively, a sustainable protocol was recently disclosed by Huang *et al*. to construct *β*-keto sulfonyl fluorides (**VI**) via inexpensive graphite felt electrodes instead of a sacrificial anode [49]. The vinyl triflates as carbon precursors combined with FSO_2_Cl allowed access to previously limited cyclic skeleton-linked sulfonyl fluorides.

**Figure 12.**
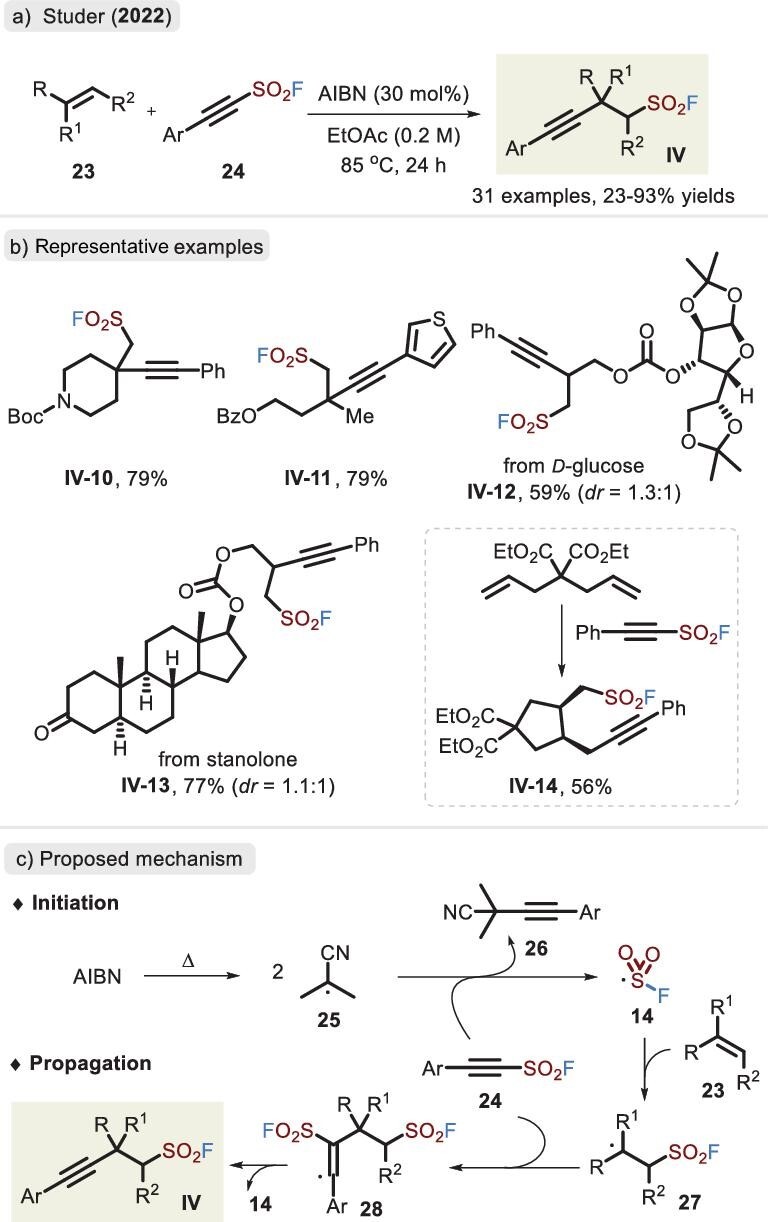
(a) Synthesis of *β*-alkynyl-fluorosulfonylalkanes via the alkenes and alkynyl sulfonyl sulfones. (b) Representative examples. (c) Proposed mechanism for the radical 1-fluorosulfonyl-2-alkynylation, adapted from [51].

The fluorosulfonyl radical, unlike the trifluoromethylsulfonyl radical [50], is not liable to release SO_2_; rather, it remains stable and manifests favorable reactivity. In 2022, the direct insertion of unactivated alkenes into alkyne sulfonyl fluorides was illustrated by Studer *et al.* (Figure 12a) [51]. This method afforded a chain of *β*-alkynyl-fluorosulfonylalkanes in good yields. However, styrene was not suitable for this reaction due to the relatively low reactivity of the benzylic radical toward compound **24**. Mechanistically, it was postulated that the homolysis of AIBN (2,2'-azobis(2-methylpropionitrile)) initiates the reaction. Subsequent radical addition of substrates **23** and **24** and *β*-elimination of radical **14** release corresponding products **IV** (Figure 12c). Unfortunately, there were considerable difficulties in the assembly of alkyne sulfonyl fluorides **24** [52], limiting the wide application of this approach.

**Figure 13.**
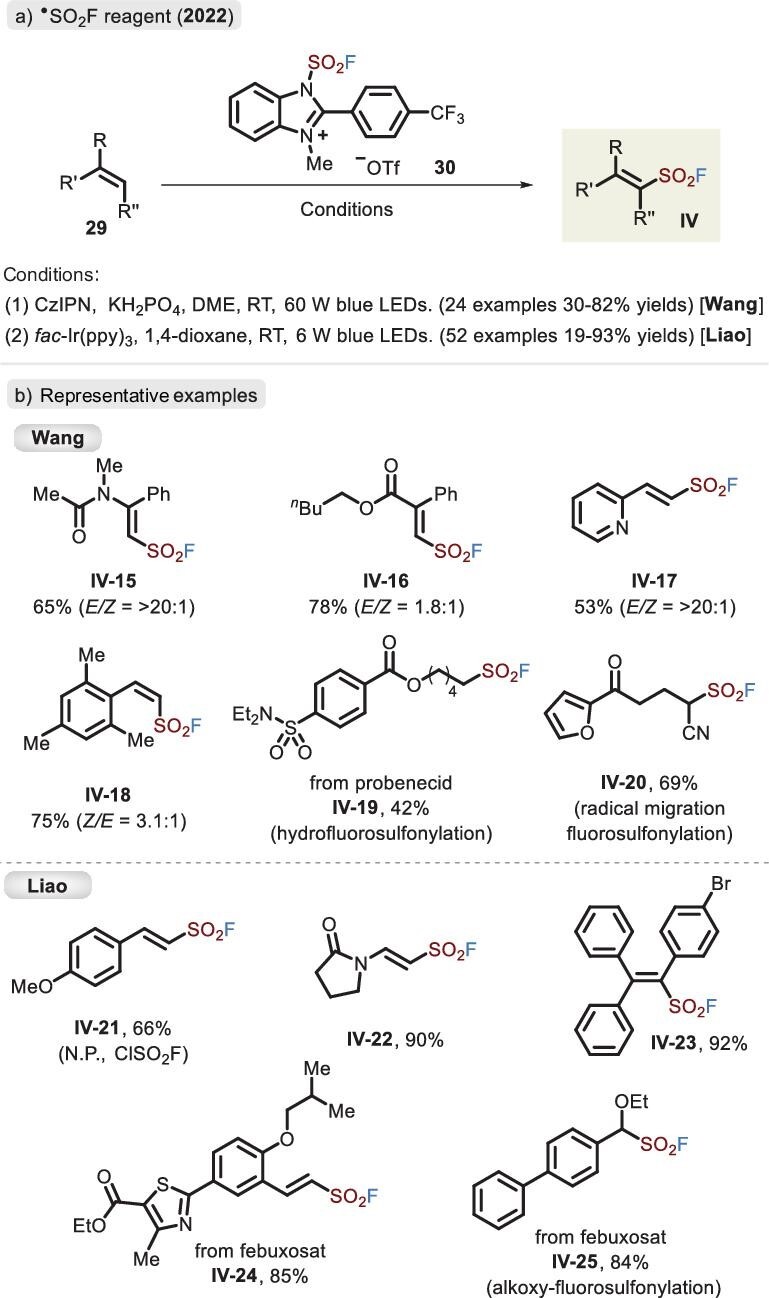
(a) Assembly of sulfonyl fluorides via the imidazolium-based fluorosulfonyl radical reagent. (b) Representative examples developed independently by the Wang and Liao groups.

Clearly, direct attachment of the fluorosulfonyl group to the carbon motif represents a concise and effective strategy, especially by the ^•^SO_2_F linker. However, the development of precursors for radical fluorosulfonylation faces several issues. ClSO_2_F, a low-boiling compound (7°C), is inconvenient to store and apply, and because of the high activity of the S–Cl bond, it easily undergoes chloride transfer to *β*-radical sulfonyl fluoride, resulting in the inaccessible trapping of other reagents. Although alkynyl sulfonyl fluoride can deliver SO_2_F radicals, it has synthetic difficulty and limited scope. Hence, in 2022, Wang *et al*. developed an analogous bench-stable reagent, imidazolium sulfonyl fluoride salt (IMSFs, **30**) [53] based upon their previous work [54]. In comparison to the FSITs (**7**) reported by Dong *et al*., its benzo and 2-aryl fragments enabled it to accept an electron, thus favoring ^•^SO_2_F release by aromatization and reacting with various alkenes (Figure 13a, conditions 1). A range of substrates involving aryl-, amido- and ester-substituents were competent, affording *E*-alkenylsulfonyl fluorides in moderate yields with excellent stereoselectivity (Figure 13b). Replacement of CzIPN with an Ir photocatalyst generated thermodynamically less favorable *Z*-type products, probably by olefin isomerization. Interestingly, hydrofluorosulfonylation of alkenes via a hydrogen source (1,4-cyclohexadiene) and migratory fluorosulfonylation from a 5-hydroxy alkene substrate were successfully achieved.

In the same year, Liao and co-workers also disclosed the same redox-active precursor, **30**, achieving radical fluorosulfonylation (Figure 13a, conditions 2) and alkoxyl-SO_2_F difunctionalization of olefins under mild photocatalytic conditions (6 W) [55]. The reagent, compared with ClSO_2_F [46], was compatible with diverse electron-rich styrenes and nitrogen- or oxygen-attached activated alkenes, yet low reactive olefins resulted in poor yields owing to the difficult-to-oxidize feature of alkyl radical intermediates.

### Disconnection of S–F bond

#### High-valent sulfur precursor

Due to the commercially available sulfonyl chloride, the substitution of fluoride for chloride frequently occurs during the assembly of sulfonyl fluoride, especially targeting small molecular skeletons. During this transformation, the addition of water (perhaps as a solvent) or a phase transfer catalyst (e.g. 18-crown-6) aids the dissolution of metal fluoride salt, thus promoting the release of the fluoride anion [56]. Although this exchange is rapid and results in a high yielding, sulfonyl chlorides, to varying degrees, undergo undesired side reactions, mainly including hydrolysis and reduction (to sulfinates) under the strong basicity of ‘naked’ fluoride. Therefore, it is essential to apply a mild fluoride source. Potassium bifluoride (KHF_2_), therein, as a superior reagent, was employed in the synthesis of sulfonyl fluorides with a wide substrate scope and almost quantitative yields, as shown in Sharpless's work in 2014 [3]. Notably, this reagent was efficiently performed via solvation and hydrogen bonding in the aqueous–organic two-phase system. Interestingly, Sharpless *et al*. developed an on-water route to realize the conversion of 2-chloroethanesulfonyl chloride via an aqueous, nearly saturated KHF_2_ solution. The strategy allowed the synthesis of ethenesulfonyl fluoride with almost quantitative yield on a kilogram scale [57]. However, a long-chain alkyl group such as *n*-octyl slowly underwent the chloride–fluoride exchange. In 2018, Barbasiewicz and Talko disclosed that the addition of catalyst TBAC (tetrabutyl ammonium chloride) in an aqueous or aqueous–organic system increased the reaction rate substantially [58].

Sulfonyl chlorides are typically unstable at ambient temperature, decomposing during the separation process. Thus, *in situ* liberation of these chlorides is an attractive approach. Indeed, deoxychlorination of sulfonic salts [59] or deaminochlorination of sulfonamides [60] could access sulfonyl chlorides and thereby sulfonyl fluorides, even enabling the late-stage modification of pharmaceuticals. Alternatively, the reaction of sulfonyl hydrazides in combination with Selectfluor to synthesize these fluorides also proceeded efficiently via sulfonyl cations or radicals [61]. Most recently, Oh and co-workers employed Et_3_N·3HF instead of Selectfluor as a fluoride source and realized the conversion of sulfonyl hydrazides into sulfonyl fluorides under electrochemical conditions [62]. In this process, Bu_4_NI functioned as not only an electrolyte but also a redox catalyst to facilitate the release of sulfonyl cations.

**Figure 14.**
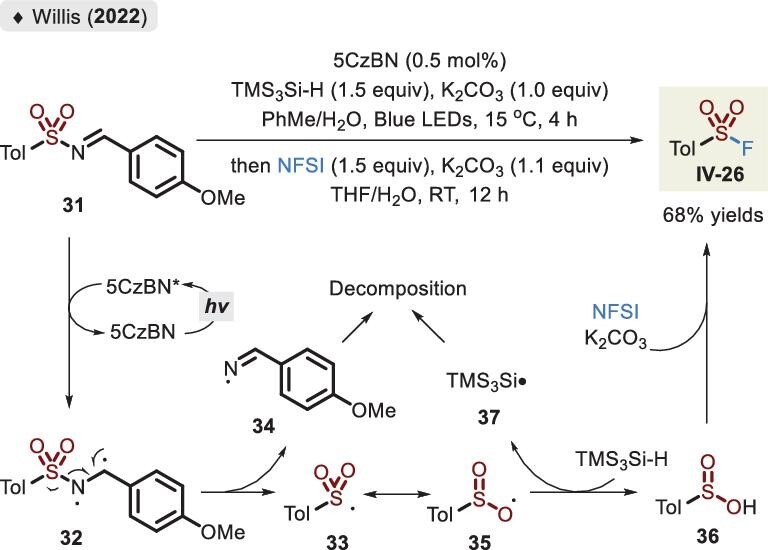
Synthesis of sulfonyl fluoride via the aldehyde-derived sulfonamide under photocatalytic conditions, adapted from [63].

In 2022, Willis *et al.* developed a complementary strategy that employed aldehyde-derived sulfonamides **31** to release radical **35** (resonance type of **33**) under photocatalytic conditions, followed by the trapping of TMS_3_Si-H, delivering sulfinic acid **36** (Figure 14) [63]. Although only an example of sulfonyl fluoride **VI-15** was synthesized via a two-step, one-pot procedure, the method was meaningful for the skeleton transformation of drug and agrochemical molecules from sulfonamide to sulfonyl fluoride. Interestingly, the photocatalyst CzBN did not undergo an electron-transfer process but offered the required activation energy from **31** to **32**.

#### Low-valent sulfur precursor

Strategies for the direct oxidative chlorination–fluorination of thiols could allow access to sulfonyl fluorides without the isolation of easily decomposed sulfonyl chlorides [64]. Therein, electrophilic fluoride, most notably Selectfluor, served as not only an oxidant but also a fluoride source and implemented the conversion of thiol derivatives such as dithiolether, sulfur ether and sulfinate [61,65]. In contrast to potassium fluoride, Selectfluor is expensive and atom-inefficient, and in most cases superstoichiometric quantities (≤7.5 equiv.) are needed, largely precluding its widespread application.

**Figure 15.**
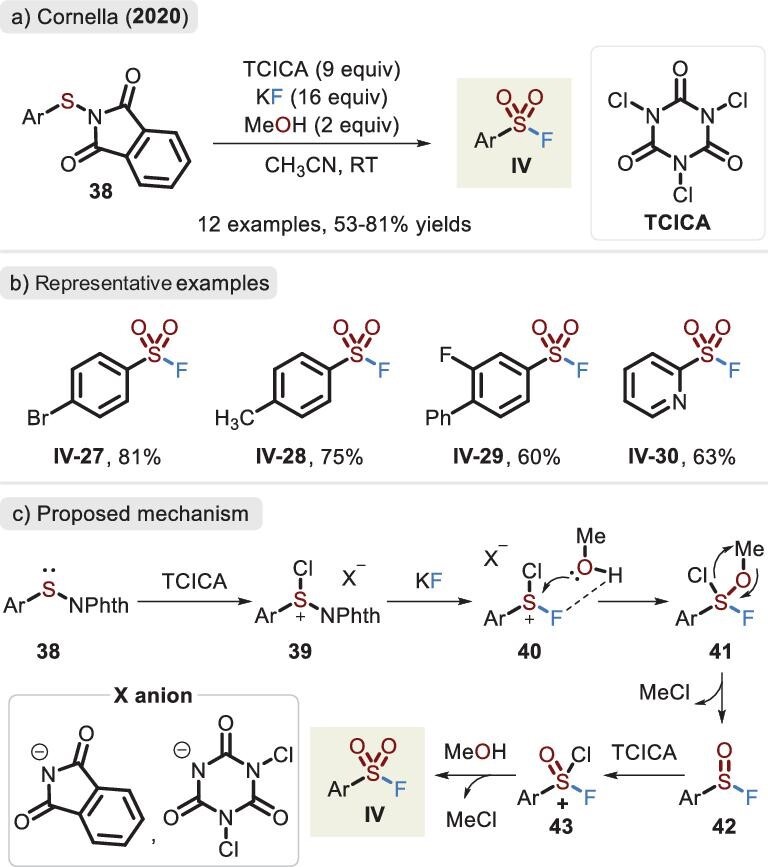
(a) Synthesis of sulfonyl fluorides via the sulfenyl phthalimide reagent. (b) Representative examples. (c) Proposed mechanism of sulfonyl fluoride, adapted from [67].

An impressive advance was disclosed in 2019 when the Noël lab reported an electrochemical oxidative protocol for the assembly of sulfonyl fluorides [66]. The route avoided the addition of oxidants, and under benign conditions involving KF as an inexpensive fluoride source, it showed good group compatibility. However, the unpleasant and unstable thiol was unavoidably used. To avoid the use of thiols, sulfenyl phthalimide as a ‘masked’ thiol precursor was introduced by Cornella and co-workers in 2020 (Figure 15a) [67]. Through fine-tuning Brønsted acids, distinct fluorinated sulfur(VI) compounds were accessed. Among them, sulfonyl fluorides were the most stable and could be isolated by using column chromatography in moderate to favorable yields. A plausible mechanism is described in Figure 15c. Initially, sulfur reagent **38** is oxidized by TCICA (trichloroisocyanuric acid), followed by replacing the Phth (or Cl) group with the fluoride anion to afford S(VI) intermediate **40**. In the presence of MeOH, the oxygen atom of protic acid then binds with the sulfur center. Upon the elimination of MeCl, sulfinyl fluoride **42** is released, which reacts again with the oxidant and acid, eventually delivering the corresponding product **IV** (analogous to the conversion of intermediate **40** into **42**). Unfortunately, the issue of excessive oxidant usage could still not be overcome; in other words, electron-rich groups were not successfully deployed.

### Disconnection of S–C and S–F bonds

#### [^−^SO_2_R] intermediate

Of the numerous available strategies, SO_2_ insertion can confer sulfonyl fluorides in the most flexible and modular fashion. Specifically, carbon-based fragments attached by sulfur centers are unhindered, avoiding the use of odorous and unfriendly thiols. Meanwhile, the reaction process does not involve the utilization of strong oxidants (e.g. Cl_2_, TCICA) and benign conditions result in excellent functional-group tolerance and accessible backbones. The emergence of stable and easily handled SO_2_ surrogates, including 1,4-diazabicyclo[2.2.2]octane-1,4-diium-1,4-disulfinate (DABSO), K_2_SO_5_, Na_2_S_2_O_4_, etc., has garnered much interest in the chemical community and considerably facilitates the introduction of hypervalent sulfur compounds. A traditionally constant modality is that upon the treatment of organic halide [68] or boride [69] with a SO_2_ surrogate, sulfinate is liberated, followed by coupling with an electrophilic fluoride in a two-step, one-pot procedure. Indeed, the development of sulfonyl fluorides via the insertion of SO_2_ is principally attributed to the earlier successful construction of sulfones and sulfonamides.

**Figure 16.**
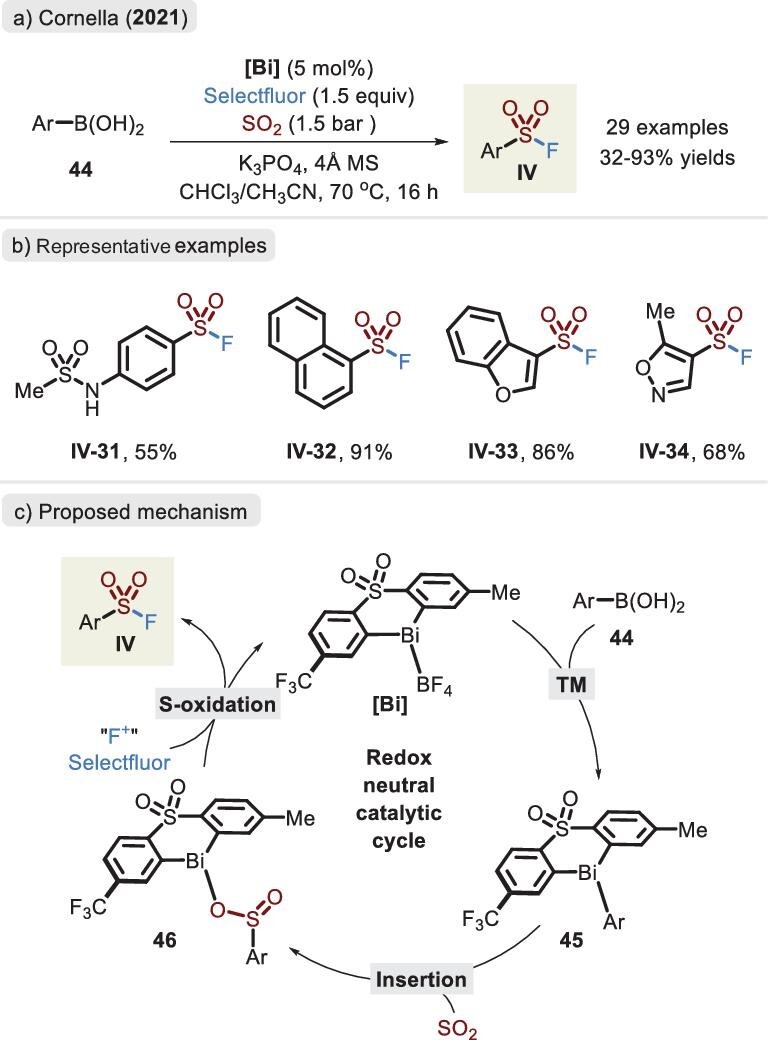
(a) Bi-catalysed one-step synthesis of sulfonyl fluorides using aryl boronic acids and Selectfluor. (b) Representative examples. (c) Proposed mechanism of sulfonyl fluoride, adapted from [70].

Notably, a meaningful result was published by Cornella *et al.* in 2021, who achieved a unique Bi-catalysed one-step assembly of sulfonyl fluorides (Figure 16a) [70]. Due to the low reactivity of the 6s^2^ lone pair in the **[Bi]** complex, this process was compatible with Selectfluor. A wide substrate scope was revealed, affording the coupling products in good yields. In contrast with the Pd-catalysed system, alkene, alkyne and bromide groups were amenable to the standard conditions and, moreover, distinct types of aromatic heterocycles effectively proceeded in this transformation. A possible catalytic cycle analogous to the essential organometallic steps was proposed (Figure 16c). The transmetalation of aryl boronic acid is first conducted via a **[Bi]** catalyst, affording intermediate **45** bearing an aryl fragment. After the insertion of SO_2_, the resulting bismuth sulfinate **46** is afforded, which undergoes the oxidation of Selectfluor to access sulfonyl fluoride **IV**. Unfortunately, when DABSO was exploited as a SO_2_ precursor, the yield of the product sharply declined.

Rongalite, or sodium formaldehydesulfoxylate dihydrate, served as a di-nucleophilic SO_2_ precursor via the release of methanal, sequentially coupling with electrophilic alkyl compounds and fluoride sources [71]. Although unfavorable alkyl thiols are avoided, the method typically requires tedious operation. To confront this limitation, a selection of strategies was disclosed by virtue of inexpensive, abundant and stable materials. In 2021, MacMillan and co-workers reported a C(*sp*^3^)–H bond functionalization for the synthesis of aliphatic sulfinic acids by decatungstate photocatalysis [72]. Upon treatment with Selectfluor, sulfonyl fluoride could be obtained, despite only an example presented with a moderate yield. In their work, a concise conversion was achieved (omitting the installation of halide group), yet the intractable problem of regioselectivity was exposed.

**Figure 17.**
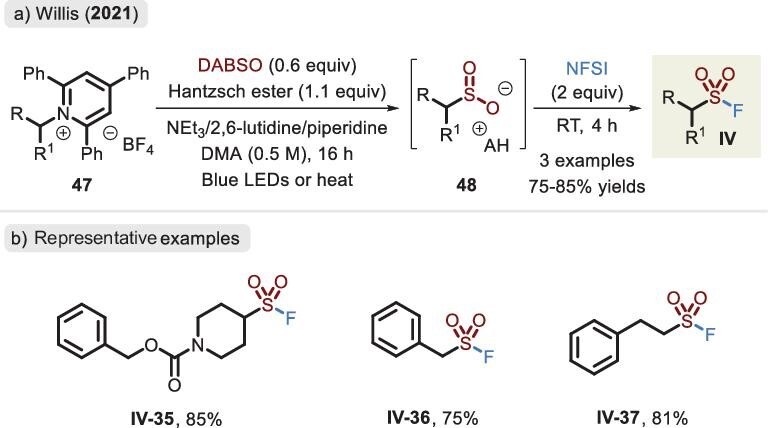
(a) Photoinduced or thermally induced process for the synthesis of sulfinates and subsequent oxidation by N-fluorobenzenesulfonimide (NFSI). (b) Representative examples of alkylsulfonyl fluoride, adapted from [73].

Interestingly, Katritzky pyridinium salts derived from commercially available amines were utilized by the Willis group as alkyl radical precursors in the assembly of fluorosulfonyl-containing compounds (Figure 17a) [73]. In this process, an electron donor–acceptor (EDA) complex was afforded, which underwent photoinduced or thermally initiated SET under catalyst-free conditions. The trapping of SO_2_ and HAT of Hantzsch ester led to sulfinate **48**. Accordingly, access to sulfonyl fluoride was readily implemented via the oxidative fluoride source.

**Figure 18.**
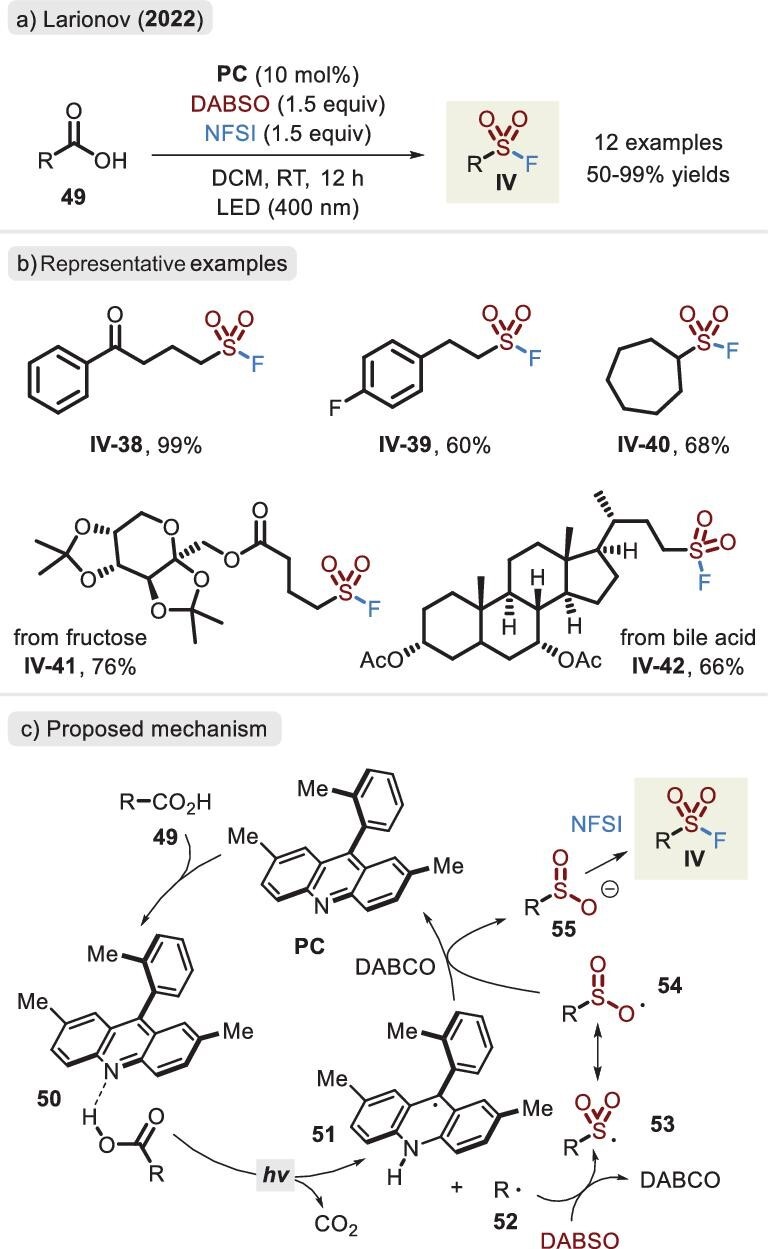
(a) Synthesis of sulfonyl fluorides via carboxylic acids under photocatalytic conditions. (b) Representative examples. (c) Proposed mechanism of the photocatalysed fluorosulfonation, adapted from [76].

The construction of sulfinate intermediates via aliphatic carboxylic acids or their derivatives and integration with electrophiles are remarkably attractive. In addition, sterically challenging molecules bearing a sulfonyl skeleton are inaccessible via traditional routes. Indeed, sodium dithionite-mediated decarboxylation of *N*-hydroxyphalimide ester (NHPI ester) effectively delivered a sulfonyl anion, thereby realizing the synthesis of sulfones, which was first established by Jiang and co-workers in 2020 [74]. Subsequently, Liu *et al.* developed an analogous protocol to access another organosulfur(VI) compound, sulfonyl fluorides, by NFSI in a one-pot, two-step format [75]. Na_2_S_2_O_4_ acted as not only a SO_2_ source but also a single-electron reductant to induce decarboxylation. Functionalized pharmaceuticals and natural products were compatible with the fluorosulfonylation process. Particularly inspiring was that aliphatic carboxylic acid could directly be a substrate, and even oxidative fluoride sources displayed favorable tolerance in a one-step procedure, which was disclosed by Larionov and co-workers (Figure 18a) [76]. Upon acridine photocatalysis, the method enabled access to a broad range of sulfonyl fluorides, including several valuable precursors, such as sulfinates and sulfonyl chlorides. Mechanistic and computational studies revealed that steric hindrance of the acridine photocatalyst plays an essential role (Figure 18c). The **PC** reagent is not susceptible to deactivation by electrophiles, and it can promote the generation of alkyl radical **52** and sulfinate **55**. The released triethylenediamine (DABCO) is responsible for **PC** entering the next catalytic cycle.

#### [^·^SO_2_R] intermediate

**Figure 19.**
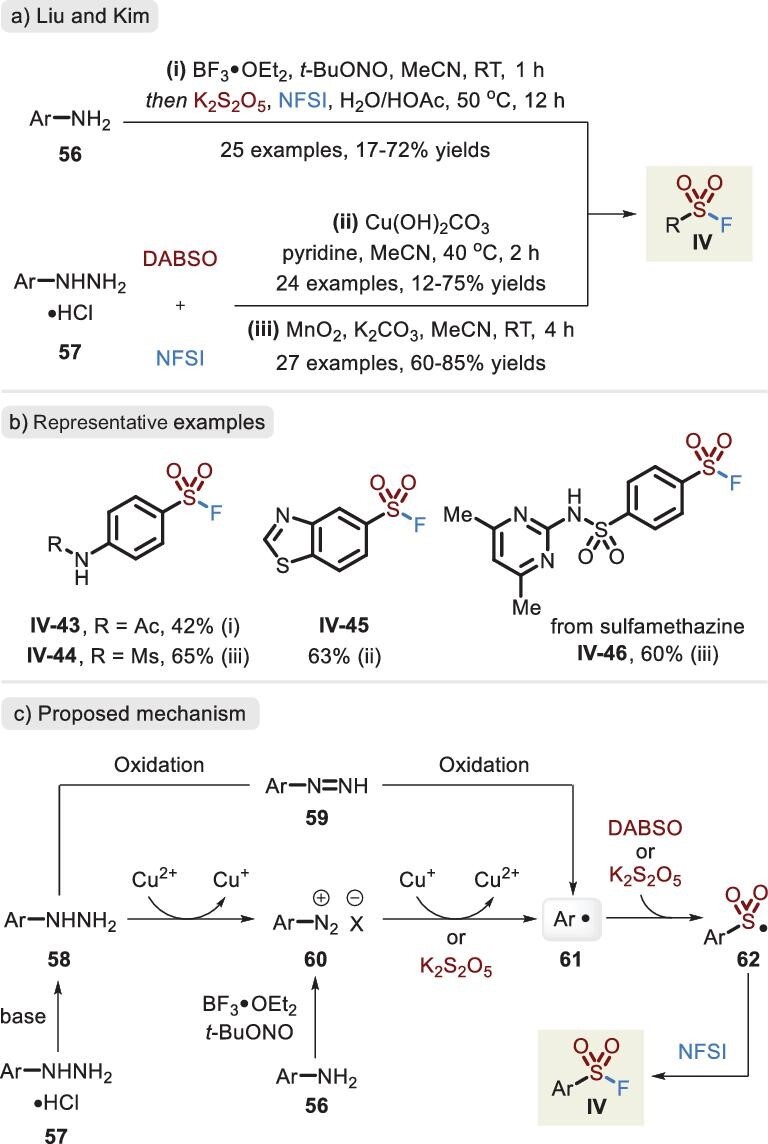
(a) Synthesis of sulfonyl fluorides from anilines or arylhydrazine hydrochlorides. (b) Representative examples by different reaction conditions. (c) Proposed mechanism via different materials and oxidants.

In contrast to sulfinate intermediates, conspicuous advantages have been achieved in the direct reaction of sulfonyl radicals with electrophilic fluorination reagents. This system permitted the presence of the oxidative fluoride in a one-step synthetic operation, thereby avoiding potential damage of reactive sulfinate. Furthermore, better reaction efficiency was observed. In 2020, a Cu-catalysed fluorosulfonylation was developed by Liu *et al*., harnessing arenediazonium salts as aryl radical precursors [77]. Soon afterward, Meng's group delineated that the treatment of diazonium salts with the oxidative fluorination reagent NFSI allowed access to arylsulfonyl fluorides [78]. The aniline-derived Sandmeyer compound (i.e. diazonium salt) is unstable and susceptible to decomposition in the course of storage. To avoid the separation loss of diazonium salt, a two-step, one-pot procedure for the synthesis of fluorosulfonyl was elaborated on by Liu and co-workers in 2022 from ubiquitous anilines (Figure 19a, condition i) [79]. Alternatively, arylhydrazine hydrochlorides, relatively stable and readily available, were effectively deployed in combination with an additional oxidant, respectively, by Liu *et al.* (Figure 19a, condition ii) [80] and Kim *et al.* (Figure 19a, condition iii) [81]. Mechanistic studies confirmed the generation of aryl radical **61** by the TEMPO-trapped complex. In Figure 19c, two possible pathways to reach intermediate **61** are presented, including (i) the reduction of arenediazonium salt **60** via the release of Cu(I) species or K_2_S_2_O_5_ and (ii) the oxidation of azo compound **59** by the additive Cu(II)/MnO_2_ or the fluorination agent NFSI. The capture of SO_2_ affords key intermediate **62**, and upon trapping by NFSI, the corresponding product is eventually afforded.

Evidently, aliphatic amine is not accommodated in the Sandmeyer-type reaction for the construction of sulfonyl fluoride because of the instability of its diazonium salt. Thus, it would be of significance to liberate an available alkyl radical. In 2017, Liu's group reported the difunctionalization of unactivated alkenes via Ag(O_2_CCF_2_SO_2_F) reagent, providing an array of *β*-trifluoromethyl sulfonyl fluorides [82]. CF_3_ and sulfonyl units are derived from the silver reagent. Afterward, *in situ* generated AgCF_3_ binding with DABSO also led to di-functional products, which were developed by the same group [83]. This showed that the transfer of one easy-to-release radical (^•^CF_3_) to another difficult-to-produce radical (*β*-alkyl radical) would expedite the assembly of modular diversity and complexity.

**Figure 20.**
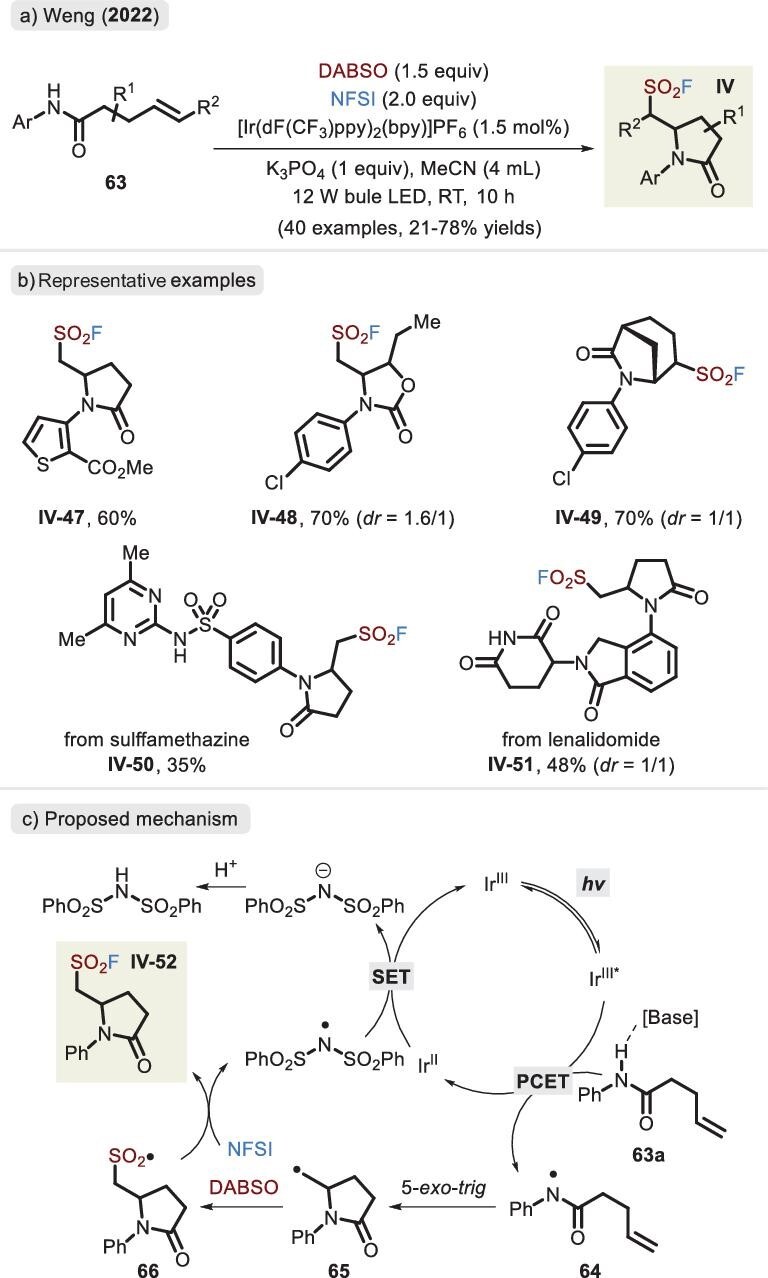
(a) Synthesis of *β*-amino-substituted sulfonyl fluorides by combining PCET with a radical transfer process. (b) Representative examples. (c) Proposed mechanism of *β*-amino sulfonyl fluoride, adapted from [84].

Indeed, the strategy disclosed by Weng and co-workers in 2022 unlocked the intramolecular aminofluorosulfonylation via the activated amidyl radical, rendering the privileged five-membered heterocycle backbone (Figure 20a) [84]. The substrate scope was broad with respect to anilines, olefins, carbamates and ureas. Notably, the endocyclic double bond was effectively geared toward the construction of a polycyclic skeleton. According to experimental results and previous reports, the process is initiated by proton-coupled electron transfer (PCET) upon photocatalysis treatment (Figure 20c). Resulting radical **65** is successively trapped by alkene and SO_2_ (via the release of DABSO), followed by oxidation of NFSI, achieving fluoride transfer and delivering *β*-amino-substituted sulfonyl fluoride. Eventually, the regeneration of the Ir(III) catalyst is accomplished via the SET process between the (PhSO_2_)_2_ N radical and Ir(II) species. Gratifyingly, the same group extended the photocatalytic system to the decarboxylative fluorosulfonylation of activated carboxylic acids [85]. It is important to mention that mechanistic and computational studies suggested an energy transfer (EnT) process rather than a SET pathway.

## HETEROATOM-LINKED FLUOROSULFOXIMINES ((F/OR^1^/NRR^1^)SO(= NR^2^)F, VI-VIII)

SOF_4_, in contrast with SO_2_F_2_, is the first multidimensional SuFEx hub and the reactive trajectory is not restricted to the plane but is tremendously flexible [26]. A range of diverse types of nucleophiles are deployed, including amines and phenols, to access sulfonimidoyl-containing compounds. In 1961, Cramer *et al*. first disclosed the synthesis of iminosulfur oxydifluorides upon treatment of SOF_4_ with primary amines [86]. The corresponding products were stable toward hydrolysis and afforded in moderate yields. Subsequently, TMS-protected sulfonimidoyl difluoride was obtained with a favorable yield from the Sundermeyer group [87]. However, SOF_4_ chemistry did not receive much attention until the SuFEx concept was developed.

**Figure 21.**
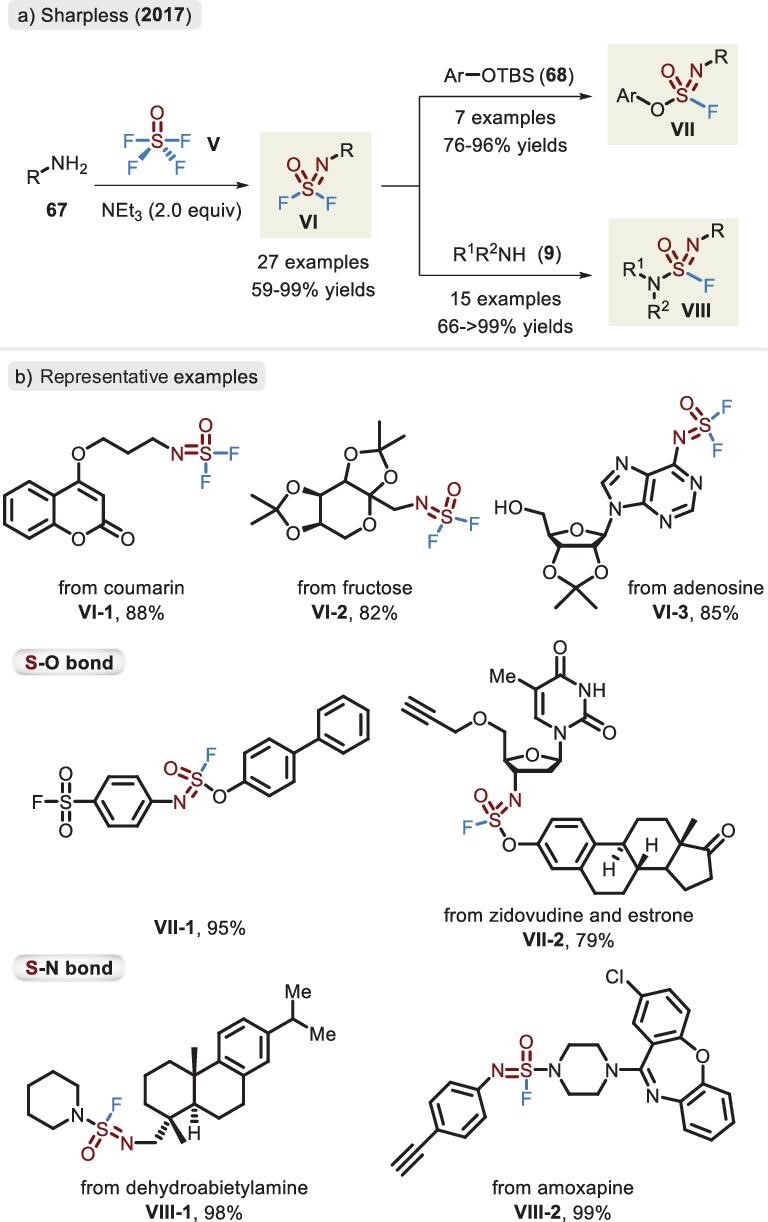
(a) Synthesis of iminosulfur oxydifluorides, sulfurofluoridoimidates and sulfuramidimidoyl fluorides via SOF_4_. (b) Representative examples for the synthesis and linkage of iminosulfur oxydifluorides, adapted from [26].

A more detailed and systematic study of SuFEx chemistry of SOF_4_ and its derivatives was conducted by Sharpless *et al.* in 2017 (Figure 21) [26]. In the presence of Et_3_N, aliphatic and aromatic primary amines smoothly reacted with saturated SOF_4_/CH_3_CN solution, rendering a library of iminosulfur oxydifluorides (**VI**) in excellent yields of ≤99%. It was notable that a distinct chemoselective propensity was observed between SOF_4_ and SO_2_F_2_: when aminophenol was exposed to the same content of both gases, aniline preferentially reacted with SOF_4_ and phenol reacted with SO_2_F_2_. Additionally, the synthetic competence of the method was further demonstrated in the assembly of complicated functional molecules. The SOF_4_-derived difluoride featuring two reactive handles could be successfully applied in the linkage of S–O and S–N bonds. Based upon TBS-activated phenols, the catalytic amount of base enabled the transformation to access sulfurofluoridoimidates **VII** in a highly effective manner. Intriguingly, Moses *et al.* employed a BTMG catalyst in combination with a HMDS additive to forge the S–O bond without installation of a silyl fragment (an analogous form presented in Figure 4) [9]. In line with SO_2_F_2_, the SOF_4_-derived substrate to produce sulfuramidimidoyl fluoride (**VIII**) was limited to secondary amines. The primary amine eventually led to an unsymmetrical sulfamide product. Competition experiments indicated that iminosulfur oxydifluorides (**VI**) display better activity toward aryl silyl ethers than SO_2_F_2_. When one S–F bond is swapped via O/N-based nucleophiles, the reactivity of the resulting compounds (**VII** and **VIII**) is dramatically weakened. The same group also developed a biocompatible SuFEx reaction via iminosulfur oxydifluorides in 2019 [88]. It effectively proceeded in the DNA and bovine serum albumin (BSA) protein linkages under aqueous buffer conditions with potential applications in chemical biology. Importantly, the SuFEx chemistry of iminosulfur oxydifluoride was applied in drug discovery via a high-throughput hit-to-lead process, reported by Sharpless, Wolan and co-workers [89]. The strategy could rapidly construct 460 diversified sulfuramidimidoyl fluoride compounds and they were directly screened to obtain drug-like inhibitors with higher activity. The same platform was also employed by Erb *et al.* in 2021, efficiently identifying an amido-imidazopyridine inhibitor based on the screen of difluoride-derived sulfamides library (nearly 300 000 small molecules) [21].

## SULFONIMIDOYL FLUORIDES (RSO( = NR^1^)F, IX)

Due to the widespread application of sulfonyl fluoride connectors, their mono-aza-bioisostere, namely sulfonimidoyl fluoride, has also started to draw considerable attention. The aza compound, interestingly, is endowed with an additional modification site, which flexibly tunes its solubility, basicity, reactivity and stability [3,4]. When the electron-withdrawing group (e.g. Ac or Ts) is installed on the nitrogen of sulfonimidoyl fluoride, increased electrophilic activity of the sulfur center was observed. In addition, relative to sulfonyl fluorides, the additional steric hindrance around sulfur renders it more stable. Molecules bearing electron-donating substituents (such as alkyl) often have remarkably low reactivity toward nucleophiles. These characteristics are also subject to the other circumstances. However, with the activation of protic acid or Lewis acid, the feedback of enhanced lone-pair electrons of nitrogen to the σ* orbital of the S–F bond enables it to be liable to defluorinate, thus releasing a highly active sulfonimidoyl cation [90].

**Figure 22.**
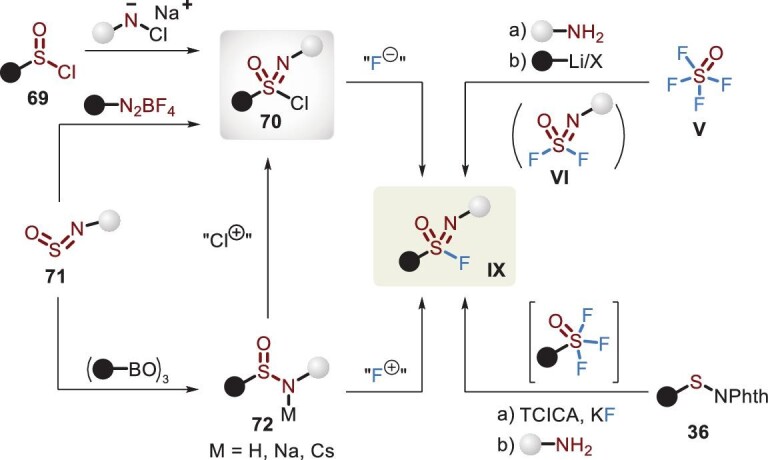
Strategies for the synthesis of sulfonimidoyl fluorides.

Since 1983, a selection of synthetic methods has been documented, as shown in Figure 22. Traditionally, the oxidative chlorination–fluorination of sulfinamide **72** allowed access to sulfonimidoyl fluoride **IX** with favorable efficiency, involving an oxidant (*^t^*BuOCl or NCS) and fluorine source (NaF, KF or tetrabutylammonium fluoride (TBAF)) [91]. The addition of a phase transfer reagent was conducive to chloride–fluoride exchange. For the sulfonyl protecting group, an available route from **69** to **IX** was alternatively described via the use of an *N*-chlorosulfonamide salt [92]. More recently, strategies for the reliable and modular assembly of sulfonimidoyl fluorides were also developed through several novel precursors, including SOF_4_ (**V**) [93], sulfenyl phthalimide (**36**) [67] and *N*-sulfinylamine (**71**) [94,95]. Furthermore, enantioenriched sulfonimidoyl fluorides could be constructed by chiral sulfinamide salt (**72**) [96].

### SOF_4_

**Figure 23.**
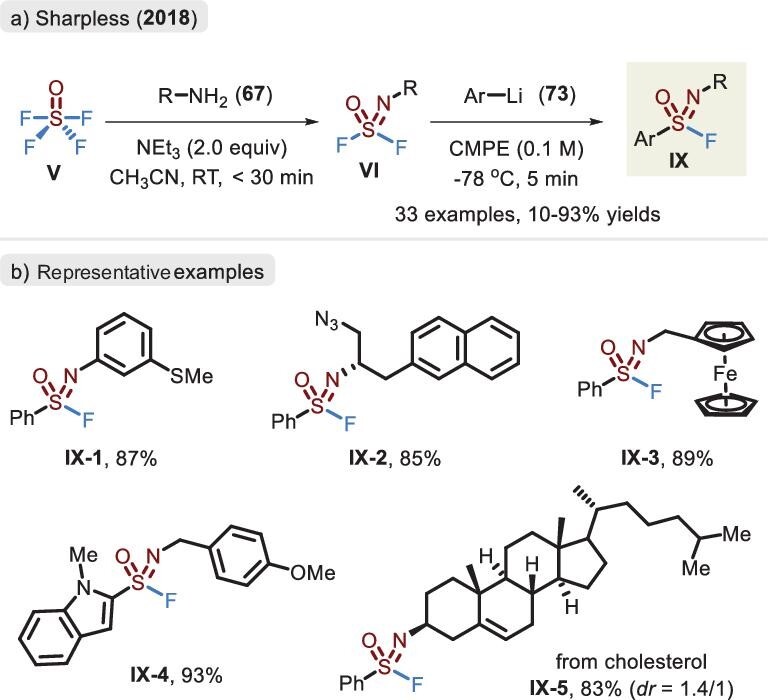
(a) Stepwise synthesis of sulfonimidoyl fluorides via SOF_4_, primary amines and aryl lithiated reagents. (b) Representative examples of sulfonimidoyl fluoride, adapted from [93].

In 2018, Sharpless and co-workers sought to extend SuFEx chemistry of SOF_4_ to carbon-based nucleophiles for controllable linkage of the S–C bond (Figure 23a) [93]. The stepwise protocol involving an isolated iminosulfur oxydifluoride and the subsequent trapping via a precast lithiated reagent provided sulfonimidoyl fluoride **IX**. Compound **VI** obtained by aliphatic or aromatic amines typically showed a favorable yield for monosubstitution. However, the electron-poor substituent (such as Ts) had a tendency toward the oversubstituted product (i.e. sulfoximine). Unfortunately, the alkyl, vinylic and alkynyl nucleophiles were poorly compatible with the current reaction.

### Sulfenyl phthalimide (RS-NPhth)

**Figure 24.**
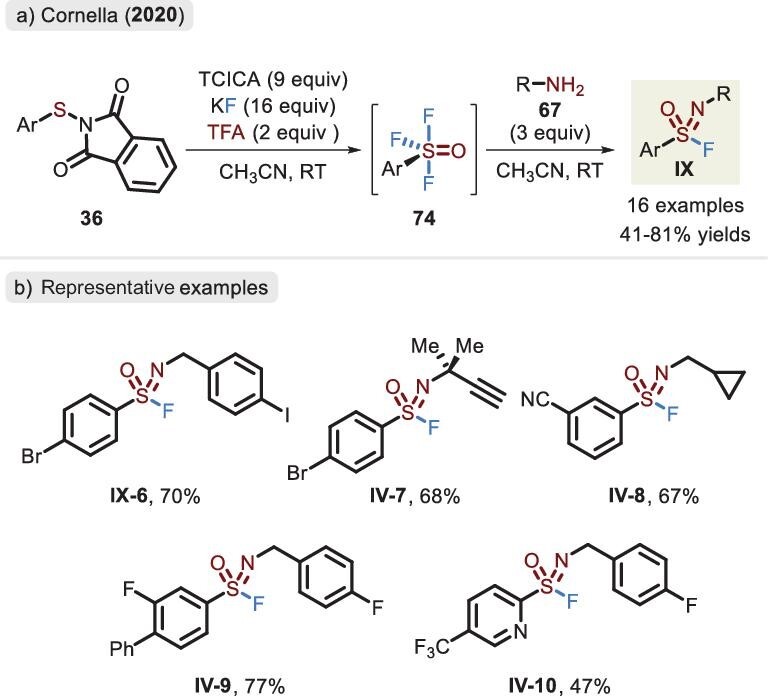
(a) Two-step synthesis of sulfonimidoyl fluorides via ArSNPhth precursors and ArSOF_3_ intermediates. (b) Representative examples of sulfonimidoyl fluoride, adapted from [67].

Interestingly, minute perturbation to reaction conditions brought about remarkable changes in the product distribution. Cornella *et al.* replaced the MeOH additive of the ArSO_2_F system (Figure 15a) with TFA and an unusual ArSOF_3_ compound (**74**) was observed with a trigonal bipyramidal geometry (Figure 24a) [67]. It exhibited strong electrophilicity and was trapped by primary amines to deliver a series of sulfonimidoyl fluorides in a two-step, one-pot protocol. The presence of water reduces the efficiency of the transformation. Therefore, the reaction process should be rigorously dried, otherwise leading to sulfonyl fluoride byproducts. In accordance with the developed ArSO_2_F system, the addition of strong oxidants (TCICA) substantially hindered the deployment of electron-rich arenes.

### *N*-sulfinylamine (RN=S=O)

**Figure 25.**
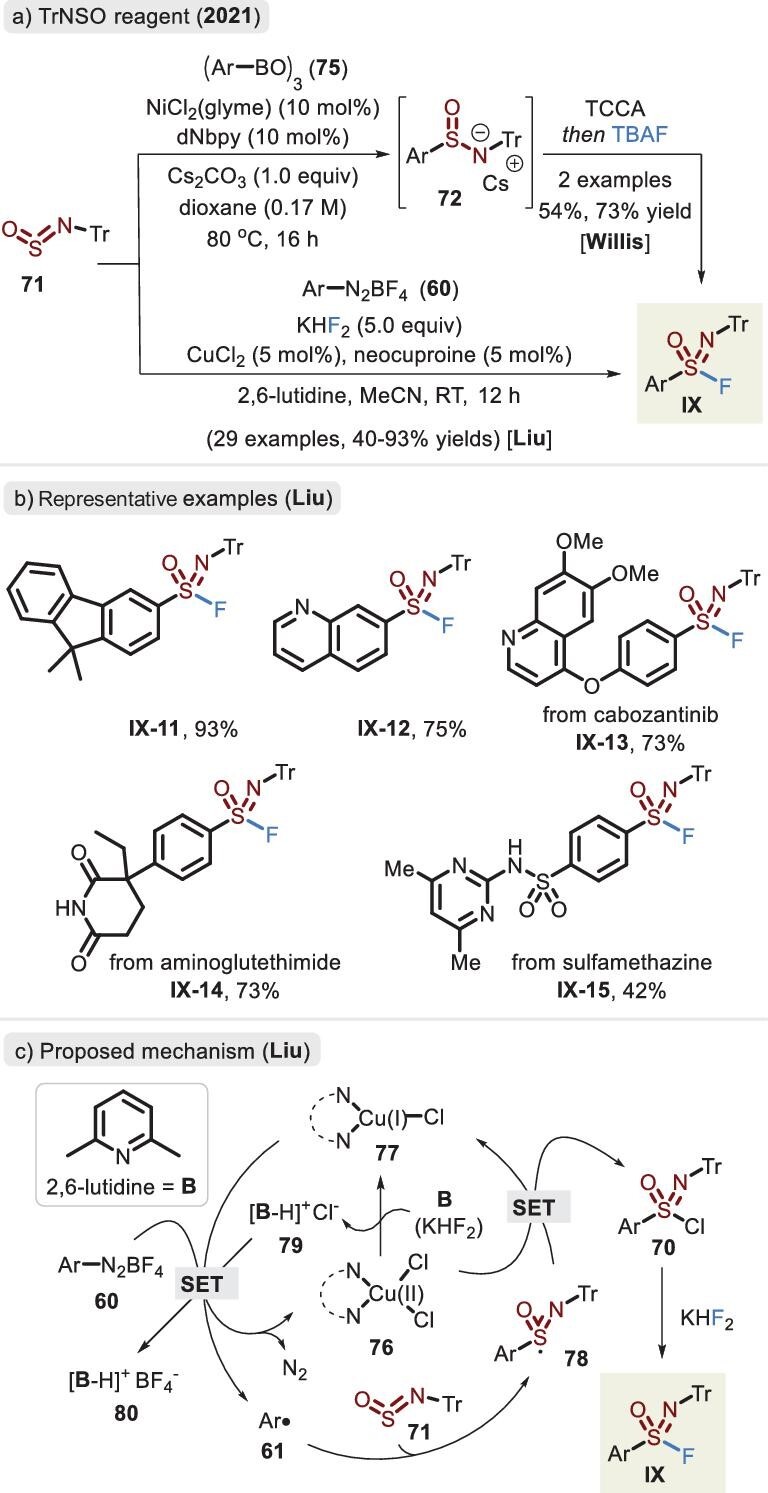
(a) Synthesis of *N*-Tr-protected sulfonimidoyl fluorides via the TrNSO reagent. (b) Representative examples of *N*-Tr sulfonimidoyl fluoride. (c) Proposed mechanism of Liu's method. (b) and (c) adapted from [95].

Inspired by the insertion of sulfur dioxide to introduce hypervalent sulfur, in 2017, Willis and co-workers developed a moisture-insensitive and readily accessible sulfinylamine reagent (TrNSO, **71**) as the N=S=O linchpin and rapidly realized the assembly of sulfonimidamides (aza analogs of sulfonamides) [97]. Afterward, the analogous protocol was disclosed by the same group based upon a benign condition. The one-pot procedure involved a Ni-catalysed sulfinamide synthesis via aryl boroxine and TrNSO in series with a classic oxidative chlorination–fluorination route [94]. Alternatively, a Cu-catalysed multicomponent reaction of arene diazonium salt, TrNSO and KHF_2_ was established by the Liu group (Figure 25a) [95]. Variation of substrates was flexible and a wide range of aniline-derived compounds proceeded to provide the desired products in moderate to favorable yields. Analogous to the reported ArSO_2_F process [77], a plausible mechanism was described herein (Figure 25c). The reduction of complex **76** to **77** via 2,6-lutidine (**B**) first provides species **79**, followed by a SET route to release the aryl radical (**61**). Meanwhile, the chloride salt **79** facilitates the regeneration of complex **76**. Then, radical **78** is efficiently liberated via the coupling of aryl radical **61** with TrNSO linchpin **71**. Resulting radical **78** undergoes a SET process via Cu(II) complex **76** to deliver intermediate **70**. Finally, upon attack of a nucleophilic fluoride source, *N*-Tr-protected sulfonimidoyl fluoride **IX** is afforded.

### Chiral sulfinamide salt

**Figure 26.**
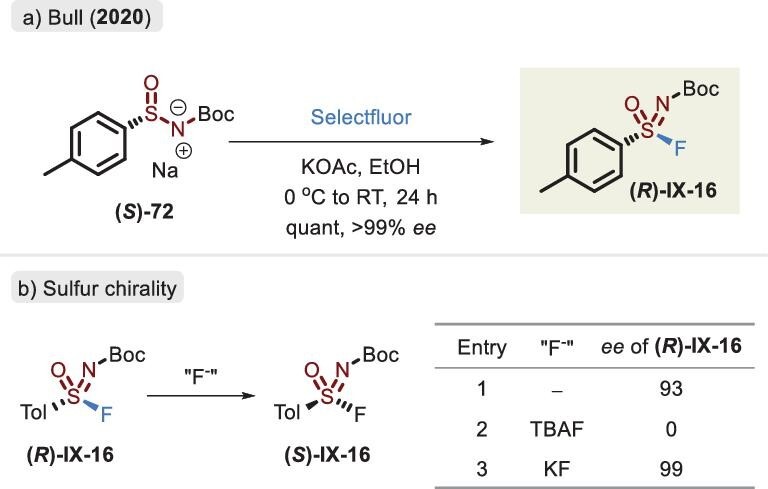
(a) Synthesis of chiral sulfonimidoyl fluoride by enantioenriched sulfinamide salt and Selectfluor. (b) Chiral retention of racemization of the enantioenriched sulfonimidoyl fluoride, adapted from [96].

Stereochemistry plays a prominent role in the biological world. The binding of a specific direction between a chiral molecule and receptor can engender a pharmacological effect. Therefore, the construction of enantioenriched SuFExable compounds is essential for drug discovery and biological probes. In 2020, an optically pure sulfonimidoyl fluoride was first isolated via chiral high performance liquid chromatography (HPLC) in the laboratory of Zuilhof [8]. Subsequently, Bull *et al.* capitalized on enantioenriched sulfinamide salt in combination with Selectfluor to afford the corresponding enantiopure product (Figure 26a) [96]. The solvent and base greatly contributed to the retention of *ee* values. In the subsequent SuFEx reaction with aliphatic amines, the near-complete racemization of the linkage compound was observed. It was supposed that the phenomenon was caused by the reattack of leaving the fluoride anion. When **(*R*)-IX-16** was exposed to a soluble fluoride source (TBAF), the *ee* values completely disappeared. The insoluble KF instead retained the stereogenic sulfur center (Figure 26b). Of particular note is that the *N*-substituted fragment influenced the optical purity of chiral sulfonimidoyl fluorides [98]. Because of the tedious preparation steps of chiral sulfinamide salt, a limited scope was achieved.

## SULFONDIIMIDOYL FLUORIDES (RS( = NR^1^)( = NR^2^)F, X)

**Figure 27.**
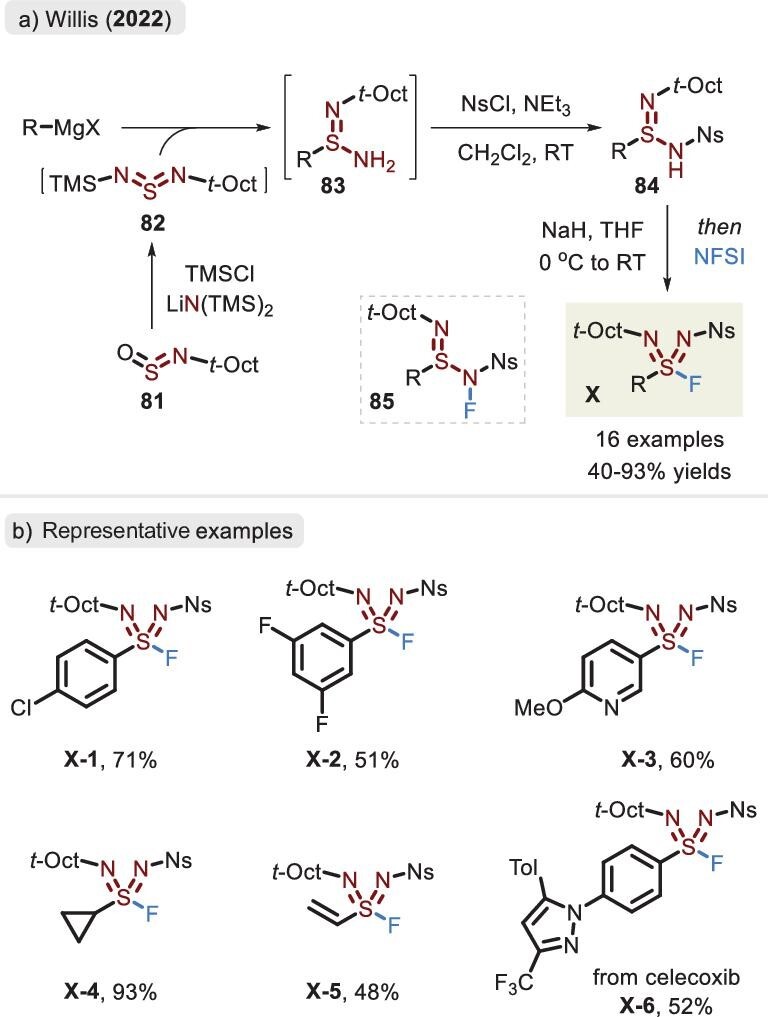
(a) Synthesis of sulfondiimidoyl fluorides commenced with the *t*-octylsulfinyl-amine reagent. (b) Representative examples of sulfondiimidoyl fluoride, adapted from [8].

The further displacement of oxygen by nitrogen in the structure of sulfonimidoyl fluoride would evolve to a novel aza analog, known as sulfondiimidoyl fluoride. Since the two vacant positions on the nitrogen can be occupied by different fragments, the diaza sulfonyl fluoride is therefore characterized by structural diversity and variable properties, showcasing a potential application in SuFEx chemistry. Although the diaza compound emerged several decades ago [99], a lack of a reliable and modular strategy hindered its uptake. In 2022, a breakthrough in the synthesis of sulfondiimidoyl fluorides was achieved by Willis *et al*. via the reaction of isolated a *N*-Ns, *N*-*t*-octyl sulfinamide intermediate and NFSI (Figure 27a) [8]. A good substrate scope was demonstrated with yields ranging from 40% to 93% and, furthermore, the products could successfully realize the coming S–N bond linkage via the activation of Lewis acids. Compound **84** was furnished through a multistep procedure: the process commences with the oxygen–nitrogen exchange of *t-*octylsulfinylamine **81**, which allows access to molecule **82** featuring the N=S=N core. In the subsequent step, the attack of the Grignard reagent delivers unstable intermediate **83**. Upon rapid extraction, the installation of the Ns group in the presence of Et_3_N is finally within reach. Of particular interest is the transformation of sulfinamide **84** to a mixture of *S*-fluorinated product and *N*-fluorinated isomer. However, the mixture needs to be left for 1–8 days at ambient temperature under air and the latter compound **85** can undergo tautomerization to evolve the former product **X**.

## CONCLUSIONS

Organosulfur(VI) fluoride compounds display high chemical stability and can efficiently achieve diverse linkages via the activation of specific conditions. On the basis of its unique nature, Sharpless and co-workers in 2014 introduced SuFEx chemistry as a new generation click reaction. Since then, SuFEx has become an active research area [100] and is frequently utilized in various fields, including organic synthesis, material chemistry, chemical biology and drug discovery. Importantly, a selection of SuFEx linkers in this process has been developed, among which fluorosulfonyl-containing molecules occupy a predominant position. Relative to the corresponding chlorides, S(VI)–fluoride compounds are not susceptible to hydrolysis and reduction. The reaction of SO_2_F_2_ with available phenols and amines affords fluorosulfates and sulfamoyl fluorides, respectively. Alternatively, solid ‘^+^SO_2_F’ precursors were synthesized with good reactivity and chemoselectivity, even enabling access to base-sensitive monosubstituted sulfamoyl fluorides. The strategies for the assembly of sulfonyl fluorides are ample and diverse, and are divided into three bond-breaking modes, including the S–C bond, S–F bond and both. Traditional routes mainly depend on chloride–fluoride exchange and chloride substrates can be directly obtained or prepared *in situ*. A new reaction pattern of the ^•^SO_2_F species was disclosed, implementing the S–C bond linkage under benign conditions. Of note, the insertion of SO_2_ to forge sulfonyl fluorides skirted unpleasant thiols, thus enabling varying of attached carbon fragments to be more flexible. In addition, photocatalysis and electrocatalysis were recently involved in a safe and green manner for the construction of S(VI)–fluoride compounds. The development of SO_2_F-containing compounds has considerably spurred their aza analogs into the SuFEx click reaction. Significantly, an additional handle on the nitrogen is offered, which expediently adjusts the property of sulfur(VI) fluorides. SOF_4_ was developed as the first 3D SuFEx linker. Upon treatment with primary amines, iminosulfur oxydifluorides were released. The SOF_4_-derived difluorides, similar to SO_2_F_2_, were able to readily forge the S–O and S–N bonds and were additionally extended to the S–C bond via lithium reagent. Other precursors, such as ArSNPhth and TrNSO, were reported and accessed mono-aza sulfonyl fluorides. The oxidative chlorination–fluorination protocol is still the most common method for the synthesis of sulfonimidoyl fluorides. A notable recent advance is the sulfondiimidoyl fluorides or diaza analogs of sulfonyl fluorides. These novel groups have already been exploited in SuFEx reactions to generate novel bioisosteres.

Despite remarkable progress in the assembly of these SuFEx connectors, the established methods still suffer from several limitations: (i) access to heteroatom-linked sulfur(VI) fluorides relies on poisonous gases, especially SOF_4_, presenting an obstacle to their widespread application; (ii) expensive oxidative fluoride sources commonly occur in the synthesis of sulfonyl fluorides, with poor atom efficiency and functional-group tolerance; (iii) the assembly of sulfonimidoyl fluorides generally originates from sulfur-bearing substrates (notably sulfinamides) and is inaccessible to obtain complex molecular architectures; (iv) chiral SuFEx connectors with the S=N fragment are less explored and only a few examples are disclosed for providing enantioenriched sulfonimidoyl fluorides via optically pure substrates. Accordingly, it is essential that safer, more efficient and modular protocols be developed to enable access to these linkers. The abovementioned hurdles, we believe, will be well addressed in the near future and other types of linkers are expected to emerge to further facilitate the development and application of SuFEx chemistry.
